# Role of genetic heterogeneity in determining the epidemiological severity of H1N1 influenza

**DOI:** 10.1371/journal.pcbi.1006069

**Published:** 2018-03-21

**Authors:** Narmada Sambaturu, Sumanta Mukherjee, Martín López-García, Carmen Molina-París, Gautam I. Menon, Nagasuma Chandra

**Affiliations:** 1 IISc Mathematics Initiative, Indian Institute of Science, Bangalore, Karnataka, India; 2 Department of Applied Mathematics, University of Leeds, Leeds, United Kingdom; 3 Computational Biology and Theoretical Physics groups, The Institute of Mathematical Sciences, Chennai, Tamil Nadu, India; 4 Homi Bhabha National Institute, Training School Complex, Anushaktinagar, Mumbai, Maharashtra, India; 5 Department of Biochemistry, Indian Institute of Science, Bangalore, Karnataka, India; National Institutes of Health, UNITED STATES

## Abstract

Genetic differences contribute to variations in the immune response mounted by different individuals to a pathogen. Such differential response can influence the spread of infectious disease, indicating why such diseases impact some populations more than others. Here, we study the impact of population-level genetic heterogeneity on the epidemic spread of different strains of H1N1 influenza. For a population with known HLA class-I allele frequency and for a given H1N1 viral strain, we classify individuals into sub-populations according to their level of susceptibility to infection. Our core hypothesis is that the susceptibility of a given individual to a disease such as H1N1 influenza is inversely proportional to the number of high affinity viral epitopes the individual can present. This number can be extracted from the HLA genetic profile of the individual. We use ethnicity-specific HLA class-I allele frequency data, together with genome sequences of various H1N1 viral strains, to obtain susceptibility sub-populations for 61 ethnicities and 81 viral strains isolated in 2009, as well as 85 strains isolated in other years. We incorporate these data into a multi-compartment SIR model to analyse the epidemic dynamics for these (ethnicity, viral strain) epidemic pairs. Our results show that HLA allele profiles which lead to a large spread in individual susceptibility values can act as a protective barrier against the spread of influenza. We predict that populations skewed such that a small number of highly susceptible individuals coexist with a large number of less susceptible ones, should exhibit smaller outbreaks than populations with the same average susceptibility but distributed more uniformly across individuals. Our model tracks some well-known qualitative trends of influenza spread worldwide, suggesting that HLA genetic diversity plays a crucial role in determining the spreading potential of different influenza viral strains across populations.

## Author summary

Levels of immunity to strains of H1N1 influenza can vary, depending on the individual. This strongly influences how the disease spreads in a population. Accounting for such variations is a major challenge for the epidemiology of infectious diseases. We study the effect of population-level genetic heterogeneity on the epidemic spread of different strains of H1N1 influenza. We model the immune response of specific ethnicities to a number of H1N1 viral strains, using this information to study disease spread for these (ethnicity, viral strain) epidemic pairs. Our results show that larger genetic diversity at the level of immune response, leading to the presence of susceptibility sub-populations with a broad distribution of susceptibilities, protects against the spread of influenza in a population. We also show that populations with a small number of highly susceptible individuals, but with a large number of less susceptible ones, should exhibit smaller outbreaks than populations with the same average susceptibility but where it is more uniformly distributed. Our work captures some qualitative trends of influenza spread worldwide, providing a first attempt at understanding how susceptibility heterogeneities arising from variations in immune response determine disease spread in populations.

## Introduction

A central aim of epidemiological studies is to identify factors that place some populations at greater risk of contracting an infectious disease than others [1]. Such factors can be associated with each of the three legs of the “epidemiologic triad” for infectious diseases, the combination of an external causative agent, a susceptible host, and an environment that links these two together [2]. Each of these could vary across populations. However, even if the causative agent was unique and environmental factors assumed to be largely common, variations intrinsic to the host can lead to large inhomogeneities in epidemic progression across populations [1, 2]. Such variations are ignored in standard formulations of compartment models for infectious diseases, which project all properties of the host onto a small set of states describing the host status. These states are typically taken to be susceptible, infected or recovered, with respect to the progress of the disease [3].

The influenza pandemic of 2009 originated in a new influenza virus, pandemic H1N1 2009 influenza A (pH1N1), to which a large fraction of the population lacked immunity [4]. The virus responsible is thought to have arisen from a mixture of a North American swine virus that had jumped between birds, humans and pigs, with a second Eurasian swine virus that circulated for more than 10 years in pigs in Mexico before crossing over into humans [4]. This pandemic caused extensive outbreaks of disease in the summer months of 2009, across the USA, Brazil, India and Mexico, leading on to high levels of disease in the winter months. The pandemic virus had almost complete dominance over other seasonal influenza viruses and was unusual in its clinical presentation, with the most severe cases occurring in younger age groups [4].

The severity of the H1N1 2009 pandemic can be assessed in terms of the basic reproduction number (*R*_0_), a fundamental dimensionless epidemiological parameter representing the average number of secondary infections caused by a typical infectious individual in a fully susceptible population. An *R*_0_ > 1 leads to an expected exponential increase in the number of infected individuals at early times, an increase which saturates before decreasing as infected individuals recover, whereas for *R*_0_ < 1, the number of infected individuals decreases monotonically. We compile estimates of *R*_0_ values for the pH1N1 epidemic across several countries from the literature, and list them in Table 1. Substantial variation in *R*_0_ values, ranging from about 1.2 at the lower end to values of 3 and above at the upper end, is evident from this table. This variation across countries illustrates the need to account for host-specific susceptibilities to disease. The immune response of the host, modulated by prior infections and vaccinations, is usually the central factor influencing *R*_0_, although location-specific contact rates and health-seeking behaviour contribute as well. In this work, we study how the spread of influenza in a population is affected by variation in naïve host immune response.

**Table 1:** *R*_0_ values for the pH1N1 epidemic in different parts of the world, compiled from literature.

Strain	Country	Basic reproduction number (*R*_0_)	Reference
A/H1N1 (2009)	New Zealand	1.55 (95% confidence interval: 1.16 to 1.86)	[5]
A/H1N1 (2009)	USA	1.3–1.7	[6]
A/H1N1 (2009)	Iran	1.32 (95% confidence interval: 1.11 to 1.59)	[7]
A/H1N1 (2009)	India	1.45	[8]
A/H1N1 (2009)	Singapore	1.2–1.6	[9]
A/H1N1 (2009)	Canada	1.57 (urban) and 3.91 (rural)	[10]
A/H1N1 (2009)	China	1.68	[11]
A/H1N1 (2009)	Japan	2.0 to 2.6 (Early May); 1.21 to 1.35 (May-July)	[12]
A/H1N1 (2009)	Mexico	1.72 (Mexico City)	[13]

Epidemics are typically modelled through deterministic compartmental-type models, represented by coupled non-linear ordinary differential equations. The SIR model is particularly well suited for studying the spread of influenza, since H1N1 is a virus which spreads from person-to-person through contact, without requiring a vector for transmission. The lack of a long incubation period and a relatively rapid recovery makes it possible to ignore the effects of immigration and emigration, as well as of births and deaths due to natural causes [3]. Models such as the SIR model and related models typically assume that individuals in the population are all alike, which allows one to reduce the number of model parameters to be estimated from data, and leads to mathematical models that can be more feasibly studied from an analytical or computational perspective. However, increasing efforts have been devoted during recent years to assessing the impact of individual heterogeneities in disease spread [14]. These heterogeneities can be of very different nature, when considering for example populations structured in specific spatial configurations [15–19], such as households [20] or age-structured populations [15], or when there exist heterogeneous individual susceptibilities, infectivities or recovery periods due, for example, to genetic [15, 21] or behavioural [22] reasons. Network or individual-based models provide a methodology for simulating each individual as a separate entity (an agent) with a specified susceptibility, an individual-specific ability to infect others as well as a specified time to recovery, while also being flexible enough to incorporate specific interaction patterns between agents. Such models, however, typically require estimating a large number of parameters. Individual-based models come with substantial overheads in terms of computational resources. In addition, their inherent stochasticity makes extensive averaging necessary [23, 24].

A straightforward generalisation of the simplest version of the SIR model involves sub-dividing populations into smaller groups or sub-populations. Individuals in each sub-population can be considered to be homogeneous, but individuals across different sub-populations can be modelled as responding differentially to the disease, as in the models of [18–21, 25–29]. Prior work has mainly focused on the theoretical analysis of these models, and relatively few attempts have been made to incorporate clinical or biological heterogeneities known to be relevant at the individual level, into population-level epidemic models. Incorporating such individual-level immunological information into population-level epidemic models accounting for susceptibility or infectivity heterogeneities has been recently identified as a major challenge for mathematical epidemiology [30].

Both innate and adaptive immune responses are initiated when an individual is exposed to the influenza virus. The innate response induces chemokine and cytokine production. Type I interferons are among the most important cytokines produced by the innate immune response and act to stimulate dendritic cells (DCs), enhancing their antigen production. The adaptive immune system can recognise the presence of an intracellular virus and mount a response only if a molecule called the human leukocyte antigen (HLA) binds to and ‘presents’ fragments of viral proteins (epitopes) to the extracellular environment. Professional antigen presenting cells such as DCs present viral antigens to CD4^+^ T-cells through HLA class-II and to CD8^+^ T-cells through HLA class-I molecules. The CD4^+^ T-helper cells promote a B-cell response and antibody secretion. HLA class-I molecules can be found on the surface of all cells, and interact with T-cell receptors (TCRs) present on CD8^+^ T-cells [31, 32]. These cells are also called cytotoxic T lymphocytes, or CTLs.

The central role of HLA-mediated presentation of antigens in the magnitude and specificity of CTL response in infectious diseases in general [33], and in influenza A in particular [34, 35], have been well studied. A recent study shows that the *targeting efficiency* of HLA, a function of the binding score of a given HLA allele and the conservation score of a given protein, correlates with the magnitude of the CTL response, and also with the mortality due to influenza A infection [36]. These studies also show that considering a single HLA allele is insufficient to determine the strength of the CTL response [34, 37].

Each individual has 6 HLA class-I alleles, the combination of all 6 alleles being referred to as an HLA genotype. Cross-reactivity between HLA alleles can result in two individuals with completely different HLA genotypes presenting the same number of high affinity epitopes [38]. Also, some alleles correlate with stronger (HLA-A*02 [34]) or weaker (HLA-A*24 [36]) CTL response to the influenza A virus. This raises a number of questions. Does a high risk allele always correlate with a severe influenza epidemic, or can the presence of diverse HLA alleles offset this risk? Are there specific patterns of susceptibility resulting from diversity in HLA, which can confer greater protection to a population? We answer these questions by using the full HLA genotype of each individual, and with an assumption that a person who presents a larger number of high affinity viral epitopes will mount a stronger CTL immune response than one who presents a smaller number [33–37, 39–43]. We use genetic diversity in HLA alleles to inform epidemiological parameters at the population level and study their influence on the epidemiological spread of H1N1 influenza.

We assume that all other factors affecting disease spread, such as contact patterns [44], health-seeking behaviour [45] and migration [46] are uniform among all individuals in a population, and across all populations. Such factors have been studied in the literature [44–46], largely using theoretical models or data collected for small cohorts. Immunological memory of an individual is also an important aspect of the immune response, and can be affected by factors such as the strain with which an individual was first infected [47, 48], prior history of infections [48] and inherited factors [49]. For lack of data regarding these factors, the model described in this work does not incorporate age and immunological history explicitly. To offset this limitation, we focus first on H1N1 strains isolated during the 2009 pandemic, for which immunological memory and vaccination proved insufficient to curb the spread of disease [4, 50]. We mine this data for characteristics which correlate with epidemic size, and test whether these correlations hold for strains isolated in years other than 2009.

In a previous paper [51], we developed a method to group together individuals who can be expected to have a similar CTL response, using the frequency of occurrence of HLA class-I alleles and the full proteome of the pathogen. We formulated an algorithm to generate all possible HLA genotypes given the frequency of occurrence of each allele in a particular population. Algorithms available through the IEDB resource [52] were used to predict the epitopes presented by each such HLA genotype. Clustering was then carried out on these HLA genotypes based on the number of epitopes presented from *each* viral protein. In this work, we use the algorithm presented in [51] to generate HLA genotypes and thereby predict high affinity epitopes presented by each such genotype. We thus identify sub-populations of individuals with comparable susceptibility to the virus. The relevant parameter in this case is the *total* number of such epitopes presented, irrespective of the viral protein from which these epitopes originate. We cluster individuals into groups based on this information, and use the clustering results to calculate the rate at which susceptible individuals become infected. This rate can be connected to the parameter *β* which appears in the conventional compartmental SIR model, which can be used to track the progress of the epidemic through the population. The prevalence of different HLA class-I alleles in different parts of the world is available through the Allele Frequency Net Database (AFND) [53]. Each population in the AFND is given an *ethnicity* tag. We predict epidemic sizes using our model for 61 such ethnicities, as well as for 81 strains of influenza A (H1N1) virus isolated in 2009, and 85 strains isolated before or after 2009, for which the genome (and hence proteome) sequence is known [54, 55].

Our results show that if we assume that the susceptibility of a given individual is inversely proportional to the number of high affinity epitopes that this individual presents for a given viral strain, we can qualitatively reproduce some known trends of influenza spread worldwide. Moreover, although the basic reproduction number *R*_0_ for a given population and a given viral strain remains the main parameter that controls the epidemic size, other characteristics of the population can also significantly impact epidemic spread. In particular, we show that a composition of HLA genotypes which results in sub-populations with widely differing susceptibilities confers protection against the spread of influenza. Moreover, populations where most of the individuals are less susceptible but where a small sub-set of individuals is highly susceptible, are better in terms of containing the disease than populations that are otherwise configured, even if they have the same value of *R*_0_. We show that the full distribution of susceptibilities across a population is required to predict the final epidemic size, but that one can extract useful information from low order moments of this distribution. Although these results are derived from pH1N1 strains, we find that the same trends apply even for viral strains isolated before or after 2009. We also show that populations with frequent occurrence of an allele associated with high risk for one strain do not always experience severe epidemics when considering influenza strains in general. We verify these conclusions by comparisons to synthetic data.

## Materials and methods

To model epidemics at the population level, we use a deterministic SIR epidemic model. We describe a population as being formed out of a number of sub-populations. Each sub-population is defined according to their specific susceptibility to the viral strain. To define these sub-populations in practice, starting from biological data, we employ the probabilistic method developed in [51]. This method uses well-tested and benchmarked algorithms for epitope prediction [52] to predict the viral epitopes presented by individuals represented by different HLA class-I genotypes. We link these genotypes to individual susceptibility against the pathogen. We can then group individuals with comparable susceptibilities into well-defined sub-populations.

We represent different epidemic scenarios in terms of *epidemic pairs*, formed by considering both the pathogen (different influenza strains) and the specific population (in this work, ethnicities) with different sub-population structures. We then use the SIR framework to track the spread of influenza through the population. The ordinary differential equations used in the model are coded in Matlab and solved numerically using Matlab’s *ode45* solver.

### Generating HLA class-I genotypes

The frequency of different HLA class-I alleles for different ethnicities estimated through large-scale genotyping is available from public databases [53]. Each individual possesses three pairs of HLA class-I genes. One HLA-A, -B and -C allele is obtained from each parent. Provided we assume that these 6 alleles occur independent of each other, we can draw 2 genes each from the full set of possible A, B and C alleles, sampling them according to the empirically measured prevalence of that allele in the population. Each combination of 6 alleles is referred to as an HLA genotype. The likelihood of finding an individual with the exact HLA genotype generated, is given by the product of the likelihood of finding each of the 6 alleles comprising the genotype. A generated genotype is only accepted if the likelihood of finding an individual with that genotype is larger than 10^−6^.

### Forming susceptibility sub-populations

An adaptive CD8^+^ T-cell mediated immune response can only be mounted against a virus if epitopes from the virus are presented by HLA class-I molecules. The binding between the epitope and the passing CTL takes place through a receptor called the T cell receptor (TCR). Not all TCRs are capable of recognising all viral epitopes. Thus if an individual presents a large number of high affinity epitopes, it is reasonable to assume that there is an enhanced probability that one or more of these epitopes can be recognised by their TCRs. Such individuals can be argued to have low susceptibility to the virus. Conversely, the ability of the immune system to present only a small number of epitopes will reduce the chance that they can be recognised. Such individuals can be argued to be more susceptible to the viral infection. This link between HLA class-I genotypes and disease susceptibility is supported, among others, by [33–37, 39–43].

#### Predicting epitopes

For a given H1N1 influenza viral strain *V* and particular ethnicity *E* forming an epidemic pair (*E*, *V*), we predict the entire set of epitopes presented by each HLA class-I allele in that ethnicity using different algorithms available through the IEDB analysis resource [52]. A consensus of three algorithms is used: an artificial neural network [56], a stabilized matrix method [57], and a combinatorial peptide-library based method [58]. These three algorithms use very different approaches for predicting epitopes for a given HLA allele. In a study carried out by Sette et. al., all peptides with strong binding affinity, *IC*50 < 50*nM*, with their cognate allele were found to be immunogenic [59]. We restrict ourselves to predictions with high likelihood of being immunogenic by ensuring coincident prediction by all three algorithms, and by only considering epitopes with predicted *IC*50 < 50*nM*. From these results, we compute the number of high affinity epitopes presented by each individual, represented by their HLA genotype, in the population.

#### Susceptibility sub-populations

The clustering of HLA genotypes into sub-populations is carried out on the basis of the number of epitopes presented, under the hypothesis that more susceptible individuals present fewer epitopes. Thus, we cluster individuals so that individuals within the same group present a similar number of epitopes, whereas individuals from different groups present different numbers of epitopes. The susceptibility of each such group is then,

$$s_{i}\propto \frac{1}{e_{i}}$$ (1)

where *s*_*i*_ relates to the susceptibility of individuals in group *i*, and *e*_*i*_ denotes the average number of epitopes presented by the HLA genotypes belonging to sub-population *i*. A discussion of the proportionality constant is provided in the section *Estimating the proportionality constant*.

Using the number of individuals *N* in the population and the classification of genotypes in clusters, we can calculate the fraction of individuals *x*_*i*_ in each sub-population *i* ∈ {1, …, *m*}, as

$$x_{i}=\frac{\text{number}\;\text{of}\;\text{individuals}\;\text{in}\;\text{cluster}\;i}{\text{total}\;\text{number}\;\text{of}\;\text{individuals}\;\text{in}\;\text{the}\;\text{population}}.$$ (2)

All the calculations described above are for a single (ethnicity, viral strain) epidemic pair. The values of all these parameters must be recalculated for each such epidemic pair being studied, since, among others, the parameter *m* depends on (*E*, *V*).

### Mathematical model

**Fig 1:**
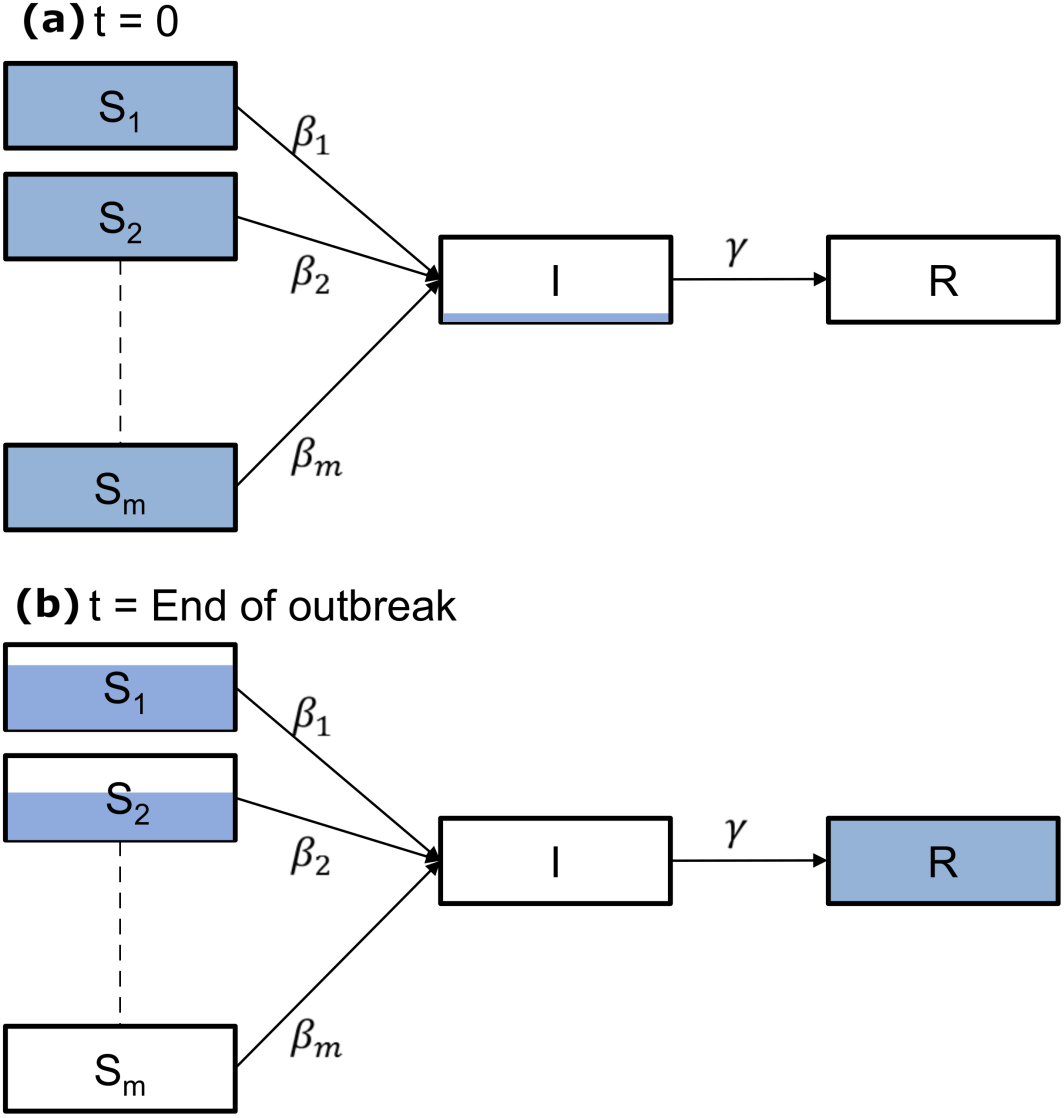
Model sketch. The SIR model with susceptibility sub-populations used in this work. (a) Initially, individuals belong to one of the susceptibility sub-populations. Infection is seeded by *a* initially infected people. (b) At the end of the epidemic, all individuals are either recovered or have never been infected.

For each epidemic pair (*E*, *V*) we use an SIR-based model to study the spread of influenza. Each population is divided into susceptibility sub-populations; see Fig 1. Our main assumptions are:

1. The population is closed and spatially well-mixed.
2. All individuals in the population have equal infectivity and recovery rates.
3. Individuals in each sub-population have the same susceptibility.
4. Individuals in different sub-populations have different susceptibilities.

We use the SIR epidemic model of [21], considering a closed population of *N* susceptible individuals and *a* initially infected individuals. The dynamics of the epidemic are represented by the coupled equations

$$\frac{dS_{i}\left(t\right)}{dt}=-\beta_{i}S_{i}\left(t\right)I\left(t\right),\;\forall i\in \left\{1,2,\ldots ,m\right\},$$ (3)

$$\frac{dI(t)}{dt}=I\left(t\right)\sum_{i=1}^{m}\beta_{i}S_{i}\left(t\right)-\gamma I\left(t\right),$$ (4)

$$\frac{dR(t)}{dt}=\gamma I(t).$$ (5)

Here *S*_*i*_(*t*), *I*(*t*) and *R*(*t*) are the numbers of susceptible (at sub-population *i*), infected and recovered individuals at time *t* and initial conditions are given by

$$S_{i}\left(0\right)=N_{i},\;\forall i\in \left\{1,2,\ldots ,m\right\},$$ (6)

$$I(0)=a\ll N_{i},\;\forall i\in \left\{1,2,\ldots ,m\right\}$$ (7)

R(0)=0, (8)

$$N=N_{1}+N_{2}+\ldots +N_{m},$$ (9)

$$N+a=\sum_{i=1}^{m}S_{i}\left(t\right)+I\left(t\right)+R\left(t\right).$$ (10)

We use *a* = 1 in our numerical calculations to represent a single infective individual who introduces the disease into a fully susceptible population.

The parameter *β*_*i*_ governing the infection of susceptible individuals belonging to the *i*^*th*^ sub-population is assumed to be a composite of three factors,

$$\beta_{i}=\alpha cs_{i}.$$ (11)

We take *αs*_*i*_ ∈ [0, 1] to represent the probability of a successful contact between a susceptible individual from the *i*^*th*^ sub-population, and an infective individual, leading to infection. The quantity *α* accounts for factors such as the infectiousness of the pathogen, or the infectivity of the infective individual, while *s*_*i*_ is related to the susceptibility of individuals in sub-population *i*. The parameter *c* represents the average number of contacts per individual per unit time. We note here that, since the dimensions of *c* are *person*^−1^*time*^−1^, *β*_*i*_ has dimensions *person*^−1^*time*^−1^. An alternative notation in the literature takes the infection rate to have units *time*^−1^, with *S* and *I* representing proportion of susceptible or infected individuals, rather than numbers. This would be equivalent to working with the alternative parameter ${\hat{\beta }}_{i}=\beta_{i}N$.

Since individuals in all the ethnicities are considered to be homogeneously mixed and all our numerical computations are carried out with the same number of individuals (*N* + *a* = 10^4^), we assume the parameter *c* to be the same for all the epidemic pairs under consideration. Further, since our interest is in analysing the impact of susceptibility heterogeneities in the spread dynamics, we take *α* to be the same regardless of the epidemic pair (*E*, *V*) under consideration. Thus, when comparing the spread dynamics between two epidemic pairs, heterogeneity in susceptibilities emerges as the main factor in our models determining the difference in these dynamics.

Finally, we note that the parameter *β*, given by

$$\beta =\frac{1}{N}\sum_{i=1}^{m}N_{i}\beta_{i},$$ (12)

can be seen as the counterpart of (*β*_1_, …, *β*_*m*_) when the population is considered homogeneous. It corresponds to the parameter widely used and estimated, usually by estimating the basic reproduction number *R*_0_, in the literature from epidemiological data for different pathogens and populations.

### Estimating the proportionality constant

For a given (*E*, *V*) pair, and using Eq (1), the susceptibility of each sub-population is inversely proportional to the average number of epitopes presented by individuals in that group. Thus we can write $s_{i}=z\frac{1}{e_{i}}$ where *z* is a proportionality constant which captures other components of the immune system that affect susceptibility, including all aspects of the innate and humoral immune response. We assume these aspects to be the same across all individuals and pairs, since only heterogeneities related to HLA profiles are considered in this work. Then, *β*_*i*_ is given by

$$\beta_{i}=\alpha cz\frac{1}{e_{i}}\;=\;y\frac{1}{e_{i}},$$ (13)

where *y* = *αcz* accounts for contributions to *β*_*i*_ that are assumed to be the same across different individuals and pairs. The value of *β* in Eq (12) can be calculated as a weighted average of the *β*_*i*_ values, as

$$\beta =\frac{1}{N}\sum_{i=1}^{m}N_{i}\beta_{i}=\frac{1}{N}\sum_{i=1}^{m}N_{i}y\frac{1}{e_{i}}=\;y\sum_{i=1}^{m}\frac{N_{i}}{N}\frac{1}{e_{i}}\;=\;y\sum_{i=1}^{m}x_{i}\frac{1}{e_{i}}.$$ (14)

The quantity *β* is henceforth referred to as *average susceptibility*. We note that our algorithm reports *e*_*i*_ = 0.07 as the minimum value of the average number of epitopes presented by a sub-population in any epidemic pair, so that *β* is always finite.

One way to obtain *y* is to scale to an experimentally determined value for *β*, given a specific ethnicity and viral strain (*E*_0_, *V*_0_). Values for *β* have historically been estimated using techniques such as serotyping the same set of people at different time points to estimate the change in the fraction of individuals susceptible to a given pathogen. Other methods are reviewed in [60]. Once we have a value of *β* for one epidemic pair (*E*_0_, *V*_0_), we can calculate values *x*_*i*_ and *e*_*i*_ for this epidemic pair using the HLA genotype generation, epitope prediction and clustering methods outlined above. These can be inserted into Eq (14), allowing us to compute the value *y*, which we have assumed to be the same across all epidemic pairs. Values of *x*_*i*_ and *e*_*i*_ for each pair (*E*, *V*) can be used, together with this value of *y*, to get a *β* for any pair (*E*, *V*).

In this work, we use the value of *R*_0_ estimated in [13] for the Mexico City population for the 2009 H1N1 pandemic originating in Mexico La-Gloria. This was chosen as a reference because HLA class-I allele frequency for this ethnicity, as well as the protein sequence of this viral strain were available. In [13], an exponential curve was fit to the data of number of infections over time during the initial phase of the epidemic. The distribution thus estimated was used to compute *R*_0_. The *R*_0_ estimated in this manner was 1.72. We use this *R*_0_ to compute *β* for this epidemic pair, and use the epitopes and sub-populations for the pair (*E*_0_, *V*_0_) = (Mexico City Mestizo pop 2, A/Mexico/LaGloria-8/2009) to estimate *y*. We note that we are using a particular *β* estimated in the literature for a specific pair (*E*_0_, *V*_0_) for computing *y*, and then considering *y* to be the same across different pairs. By doing this, we are *scaling* the rate of the *event* *S*_*i*_ + *I* → *I* + *I* in all the simulations for any pair (*E*, *V*) to the value of *β* obtained from data for the given pair (*E*_0_, *V*_0_).

### Summary statistics for comparing epidemics

We focus on the following global epidemiological characteristics:

$$\begin{array}{ccc} FI_{\infty } & = & \frac{R(\infty )}{N+a}\;=\;\text{Total}\;\text{fraction}\;\text{of}\;\text{individuals}\;\text{suffering}\;\text{from}\;\text{the}\;\text{infection}\\ & & \text{during}\;\text{the}\;\text{outbreak},\\ R_{0} & = & \text{Basic}\;\text{reproduction}\;\text{number}\;=\;\text{Number}\;\text{of}\;\text{secondary}\;\text{infections}\\ & & \text{caused}\;\text{by}\;\text{a}\;\text{typical}\;\text{infected}\;\text{individual}\;\text{in}\;\text{a}\;\text{fully}\;\text{susceptible}\;\text{population,}\\ & & \text{until}\;\text{they}\;\text{recover.} \end{array}$$

In our model, the ability of an individual to transmit the disease does not depend on the sub-population that the infected individual belongs to, since infectivity is considered to be the same across sub-populations. The SIR model of Eqs (3)–(10) was analysed in [21], where it was proved that *R*(∞) is the only positive solution of

$$N+a-R\left(\infty \right)-\sum_{i=1}^{m}N_{i}e^{-\frac{\beta_{i}R\left(\infty \right)}{\gamma }}=0,$$ (15)

and *FI*_∞_ can be derived from *R*(∞) by applying $FI_{\infty }=\frac{R(\infty )}{N+a}$. The basic reproduction number *R*_0_ is the number of secondary infections that a typical infected person causes when introduced into a large population of susceptible individuals. In the classical SIR model for homogeneous populations, *R*_0_ is given by

$$R_{0}=\frac{\beta N}{\gamma }.$$ (16)

In order to calculate *R*_0_ for our system of equations (Eqs (3)–(10)), we consider the case when a small number of infected individuals is introduced into a large population of *N* susceptible individuals. We assume the number of susceptible individuals (*S*_*i*_(0) = *N*_*i*_ for all *i*) to be large, such that *a* ≪ *N*_*i*_. This approaches the limit in which there is an unlimited source of susceptible individuals at the beginning of the epidemic. Then the dynamics of the initially infected population in terms of *a*(*t*), the number of initially infected individuals at time *t* declines as

$$\frac{da(t)}{dt}=-\gamma a(0),$$ (17)

and thus *a*(*t*) = *a*(0)*e*^−*γt*^. Let *I*^(1)^(*t*) be the number of secondary infections caused up to time *t*, with *I*^(1)^(0) = 0, by the *a* initially infected individuals. Then

$$\frac{dI^{\left(1\right)}\left(t\right)}{dt}=\sum_{i=1}^{m}\beta_{i}S_{i}a\left(t\right)\;=\;a\left(0\right)e^{-\gamma t}\sum_{i=1}^{m}\beta_{i}N_{i},$$ (18)

so that $I^{\left(1\right)}\left(t\right)=a\left(0\right)\sum_{i=1}^{m}\beta_{i}N_{i}[\frac{e^{-\gamma s}}{-\gamma }{]}_{0}^{t}$.

The basic reproduction number is given by

$$R_{0}=\underset{t\to \infty }{\text{lim}}I^{\left(1\right)}\left(t\right)\;=\;a\left(0\right)\sum_{i=1}^{m}\beta_{i}N_{i}[\frac{e^{-\gamma t}}{-\gamma }{]}_{0}^{\infty }\;=\;\frac{\sum_{i=1}^{m}\beta_{i}N_{i}}{\gamma }a\left(0\right),$$ (19)

so that by setting *a*(0) = 1 we get

$$R_{0}=\frac{\sum_{i=1}^{m}\beta_{i}N_{i}}{\gamma }.$$ (20)

For *m* = 1, this expression leads to the well-known basic reproduction number for the homogeneous case (Eq (16)).

#### Parameters characterising epidemic pairs

Our model predicts values of *FI*_∞_ and *R*_0_ for each pair (*E*, *V*). Any given epidemic pair (*E*, *V*) corresponding to an ethnicity *E* and a viral strain *V* has a *susceptibility profile* described by the number *m* of sub-populations, and by vectors (*β*_1_, …, *β*_*m*_) and (*N*_1_, …, *N*_*m*_). The *susceptibility profile* of any epidemic pair (*E*, *V*) is described by a Susceptibility Profile Vector (*SPV*)

$$SPV\left(E,V\right)=\underset{N_{1}}{\underset{︸}{(\beta_{1},\ldots ,\beta_{1}}},\underset{N_{2}}{\underset{︸}{\beta_{2},\ldots ,\beta_{2}}},\ldots ,\underset{N_{m}}{\underset{︸}{\beta_{m},\ldots ,\beta_{m})}}.$$

The quantities *FI*_∞_ and *R*_0_ can be expected to directly depend on the *SPV*(*E*, *V*), where we omit (*E*, *V*) from now on for ease of notation. For example, it is clear that for a given epidemic pair, *R*_0_ directly depends on the total number of individuals, *N*, the recovery rate, *γ*, and the average susceptibility

$$\beta =\frac{1}{N}\sum_{i=1}^{m}N_{i}\beta_{i}\;=\;E\left[SPV\right].$$

On the other hand, the quantity of central interest to epidemic modeling, the final epidemic size *FI*_∞_ for a given epidemic pair, could depend on the full distribution of the *SPV*. For concreteness, we examine the dependence of *FI*_∞_ on the lower order moments of the distribution, such as the standard deviation, the skewness and the coefficient of variation, defined respectively as

$$\begin{array}{ccc} \sigma \left(SPV\right) & = & \sqrt{Var\left(SPV\right)}\;=\;\sqrt{\frac{1}{N}\sum_{i=1}^{N}{\left(SPV_{i}-\beta \right)}^{2}},\\ Sk\left(SPV\right) & = & Skewness\left(SPV\right)\;=\;\frac{\frac{1}{N}\sum_{i=1}^{N}{\left(SPV_{i}-\beta \right)}^{3}}{{\left(\sqrt{\frac{1}{N}\sum_{i=1}^{N}{\left(SPV_{i}-\beta \right)}^{2}}\right)}^{3}},\\ CV\left(SPV\right) & = & \frac{\sigma \left(SPV\right)}{\beta }. \end{array}$$

We note that a long left tail of the distribution represented by *SPV* would result in *Sk*(*SPV*) < 0, indicating the presence of a small number of individuals with susceptibility significantly lower than the mean. On the other hand, when the population has a small representation of individuals with susceptibility significantly higher than the mean, we have *Sk*(*SPV*) > 0.

### Workflow

**Fig 2:**
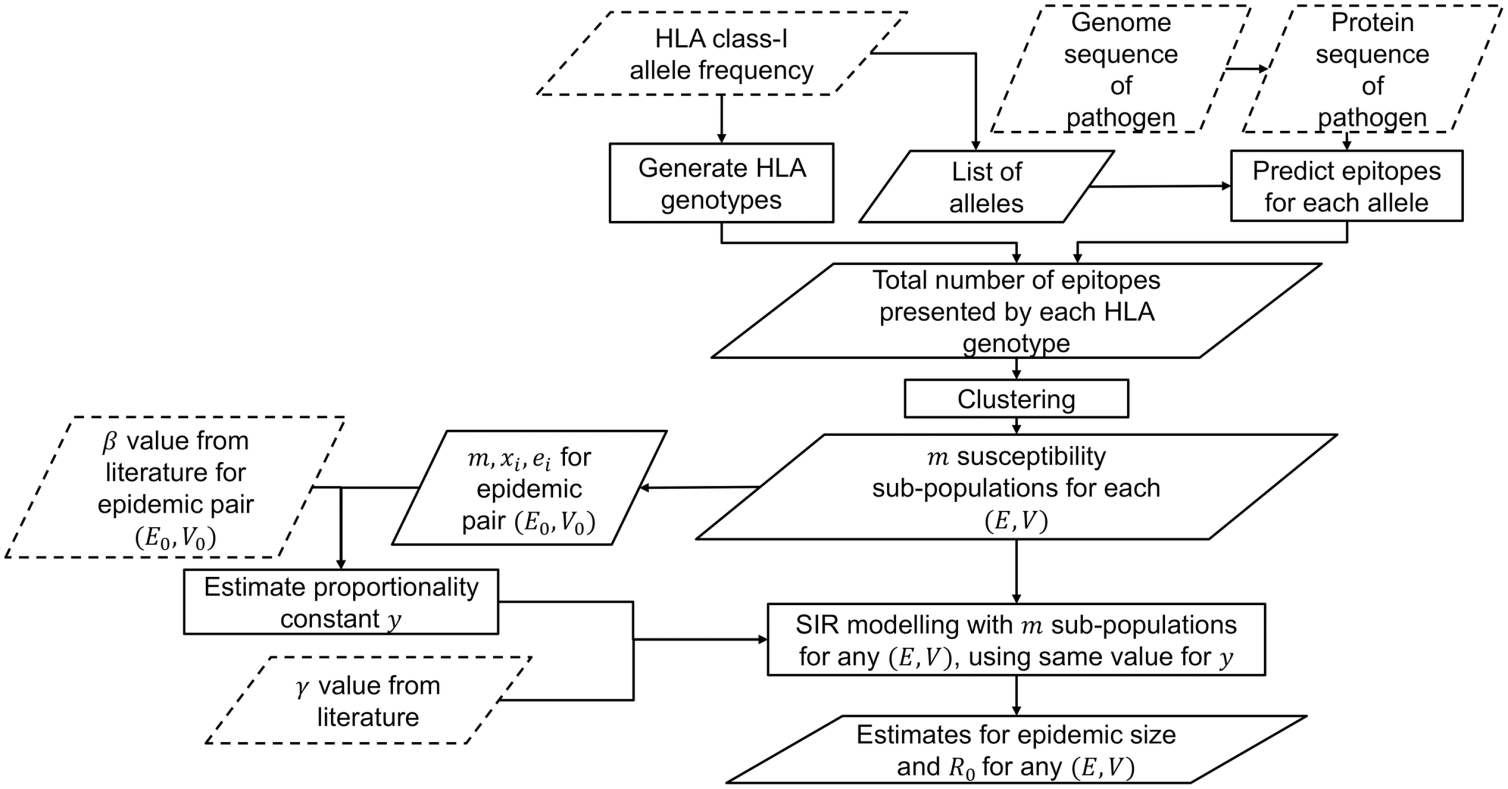
Workflow. Summary of the steps carried out in this work. Inputs from external sources are shown in dotted parallelograms.

The workflow used in this paper is summarised in Fig 2.

## Results

To compute *FI*_∞_, we solve Eqs (3)–(10) with *N* + *a* = 10^4^ individuals, *a* = 1. Each simulation is allowed to run for (0, *T*), where time *T* is large enough to ensure that the epidemic has died out. In particular, *T* is chosen to be large enough for each considered epidemic pair so that *R*(*T*) ≈ *R*(∞) obtained from the simulation satisfies Eq (15) with some error *ϵ* < 10^−2^. The recovery rate used was *γ* = 1/3 *day*^−1^ [13].

**Fig 3:**
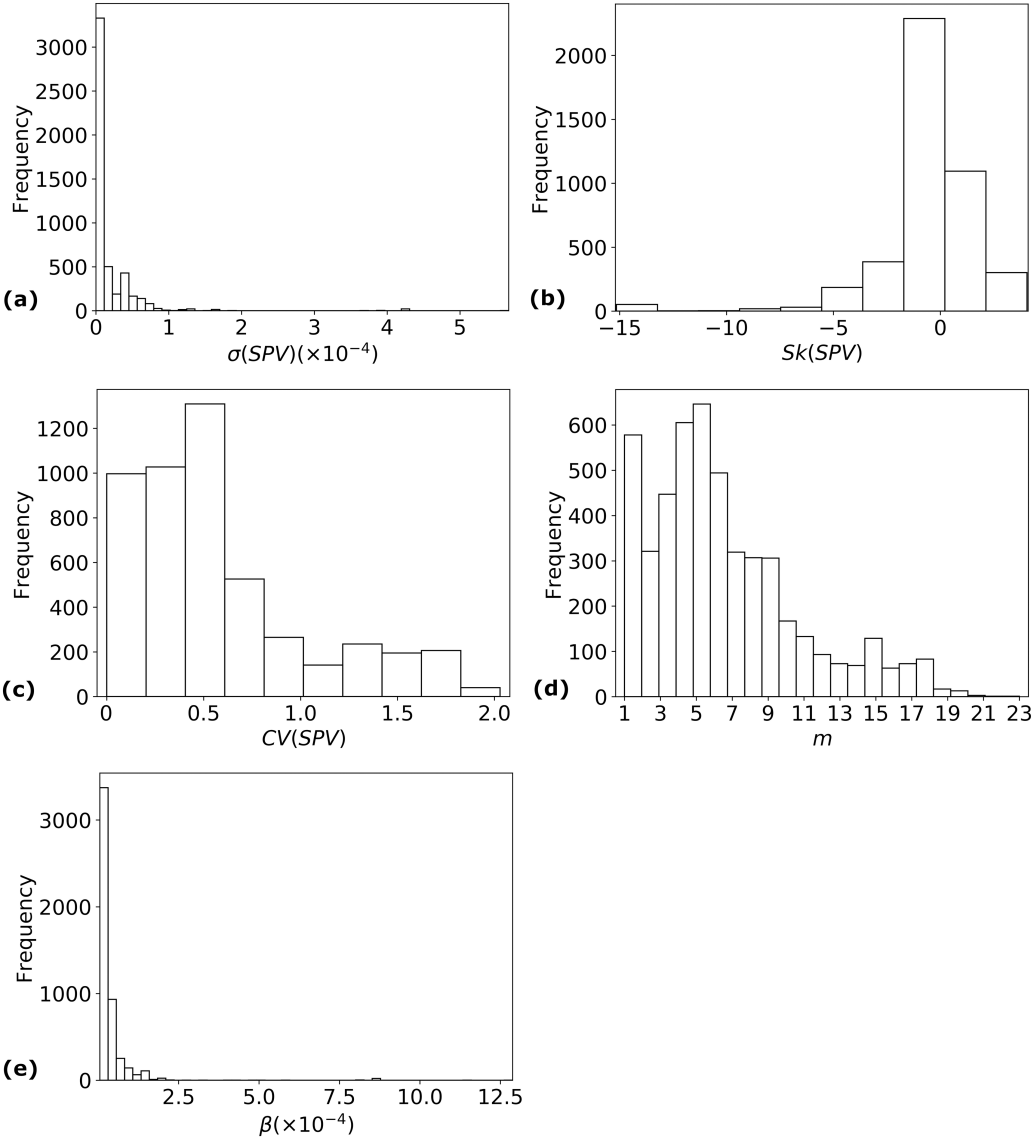
Variations in *SPV* characteristics, 2009 strains. Histograms for the values of the different susceptibility profile vector characteristics for the 4, 941 epidemic pairs involving H1N1 strains isolated in 2009: (a) *σ*(*SPV*); (b) *Sk*(*SPV*); (c) *CV*(*SPV*); (d) *m*; and (e) *β*.

The input to Eqs ((3)–(10)) was determined for 61 ethnicities and 81 viral strains isolated in 2009, leading to the study of 4, 941 epidemic pairs. Of these, 1, 392 cases had *R*_0_ > 1, and 718 cases had *FI*_∞_ > 0.5. The distributions of *SPV* characteristics across these 4, 941 epidemic pairs is provided in Fig 3. The number *m* of susceptibility sub-populations varied from 1 (578 cases) to 23 (1 case, A/Giessen/6/2009 with Kenya Nandi ethnicity). The most common value for *m* was 5, seen in 647 cases spanning 80 strains and 32 ethnicities. Details regarding ranges of calculated parameters for strains isolated before or after 2009 can be found in the supporting information; see S1 Fig. All estimated parameters are provided for all epidemic pairs in a supplementary file; see S1 File.

Results presented in upcoming sections are for H1N1 strains isolated in 2009, unless stated otherwise.

### Dependence of epidemic size and *R*_0_ on average susceptibility

**Fig 4:**
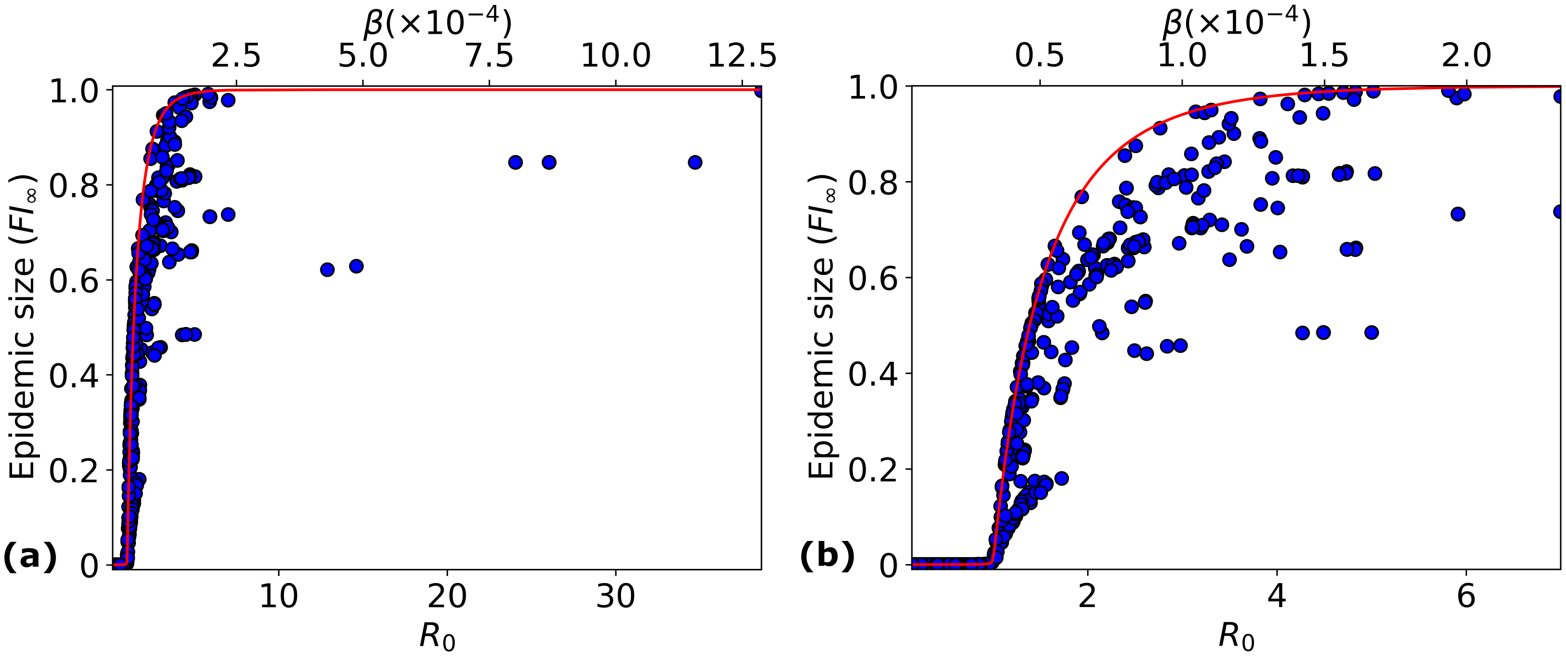
*R*_0_ cannot predict epidemic size exactly. The dependence of *FI*_∞_ on *R*_0_ and *β* is shown for: (a) all epidemic pairs involving strains isolated in 2009; (b) epidemic pairs involving strains isolated in 2009, and with *R*_0_ < 7. Only epidemic pairs with *m* > 1 are plotted. The red line shows the epidemic size in the case of homogeneous susceptibilities. We see that when *R*_0_ > 1, *FI*_∞_ takes on a wide range of values for any given *R*_0_.

We first examine the relationship between the average susceptibility (*β*), the basic reproduction number (*R*_0_) and the epidemic size (*FI*_∞_); see Fig 4. We note that Eq (16) predicts a linear relationship between *R*_0_ and *β*. As can be seen in Fig 4(a), most (*E*, *V*) pairs have *β* < 2 × 10^−4^*person*^−1^*day*^−1^, while pairs with higher values of *β* correspond to those with large epidemic sizes (*FI*_∞_ > 0.6). These pairs have *R*_0_ > 7, implying *β* > 2.33 × 10^−4^*person*^−1^*day*^−1^ from Eq (20).

In Fig 4(b) we focus on epidemic pairs with *R*_0_ < 7. In this plot, there are a large number of points with epidemic size *FI*_∞_ ≈ 0. Upon closer examination, these points turn out to have *R*_0_ < 1, as expected. We note that *R*_0_ = 1 implies *β* = 0.33 × 10^−4^*person*^−1^*day*^−1^, which corresponds to the point in Fig 4(b) where the epidemic size starts to rise above 0. In all further plots, we focus on the (*E*, *V*) pairs where 1 < *R*_0_ < 7 and *m* > 1, leading to the analysis of 956 epidemic pairs.

### No single parameter predicts epidemic size

In Fig 4(b), where the relationship between *β* and *FI*_∞_ is shown, it can be seen that a high value of average susceptibility leads to a larger epidemic. The red line corresponds to the epidemic size when the susceptibility compartment is homogeneous (*i.e.*, *m* = 1). We see that this line forms an upper bound on the *FI*_∞_ values for epidemic pairs with *m* > 1. It has been proved that the final epidemic size is always lower in an epidemic pair with heterogeneous susceptibility, than an epidemic pair with the same average susceptibility but with homogeneous susceptibility [21, 28, 44, 61]. The predictions in our simulations agree with this result. However, we observe a spread of *FI*_∞_ values when considering epidemic pairs containing heterogeneous susceptibilities and having the same average susceptibility *β*; see Eq (12). This shows that heterogeneity plays a role in determining the extent of an epidemic even when the average susceptibility remains constant.

**Fig 5:**
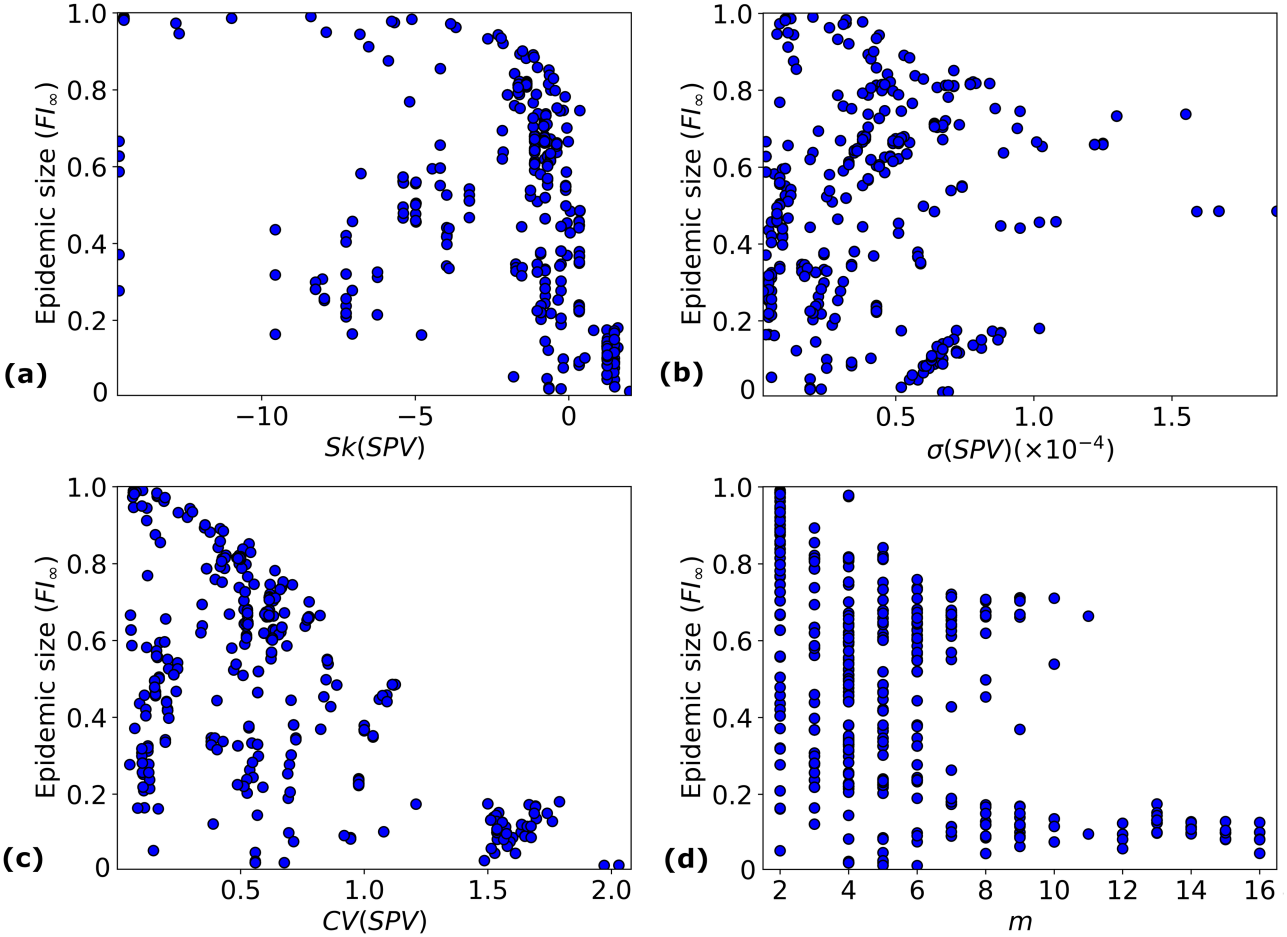
*FI*_∞_ as a function of *SPV* characteristics. The dependence of *FI*_∞_ on several characteristics of the susceptibility profile vector of each (*E*, *V*) pair involving an H1N1 strain isolated in 2009: (a) skewness of the *SPV*; (b) standard deviation of the *SPV*; (c) coefficient of variation of the *SPV*; (d) number of susceptibility sub-populations, *m*.

To study what aspects of this heterogeneity have the greatest impact on epidemic size, we examine the dependence of *FI*_∞_ on the characteristics of the susceptibility profile vector discussed above (*m*, *β*, *σ*(*SPV*), *CV*(*SPV*) and *Sk*(*SPV*)); see Fig 5. The main trends that can be identified are the following:

- Epidemic pairs leading to positive skewness of the *SPV* seem to yield smaller epidemic sizes on average; see Fig 5(a).
- Pairs corresponding to *SPV* with larger coefficient of variation also yield smaller epidemic sizes; see Fig 5(c).
- Epidemic pairs containing more sub-populations (larger *m*) correspond to small epidemic sizes; see Fig 5(d).

In our data, a positive value for *Sk*(*SPV*) corresponds to epidemic pairs with *R*_0_ < 2. Fig 4 shows that even with such small values for *R*_0_, *FI*_∞_ can take on a wide range of values, going up to 0.8. Yet, epidemic pairs in our data set with positive *Sk*(*SPV*) always have *FI*_∞_ < 0.2; see Fig 5(a). This suggests that having a positive skewness, corresponding to a distribution where most of the people have low susceptibility, but a small number of people have susceptibility significantly larger than the mean, lends some protective effect to the population.

Although *σ*(*SPV*) does not directly affect *R*_0_, it influences it indirectly due to the positive correlation between *β* and *σ*(*SPV*). To remove this correlation, one can analyse *CV*(*SPV*) instead; see Fig 5(c). This figure indicates that epidemic pairs with larger values of *CV*(*SPV*) lead to smaller epidemic sizes.

We provide correlation coefficients *r*(*θ*, *τ*) ∈ (−1, 1) between our summary statistics *τ* ∈ {*FI*_∞_, *R*_0_} and *SPV* characteristics *θ* ∈ {*m*, *β*, *CV*(*SPV*), *σ*(*SPV*), *Sk*(*SPV*)} in Table 2. The parameter *β* provides the best predictor for both *R*_0_ and *FI*_∞_. On the other hand, the heterogeneity described by *CV*(*SPV*), and the skewness of the susceptibility distribution described through *Sk*(*SPV*), also emerge as good predictors of *FI*_∞_.

**Table 2:** Correlation coefficients *r*(*θ*, *τ*) between summary statistics of the epidemic and *SPV* characteristics.

*SPV* Characteristic, *θ*	*FI*_∞_	p-value	*R*_0_	p-value
*m*	−0.51	< 10^−3^	−0.24	< 10^−3^
*β*	0.74	< 10^−3^	1.00	< 10^−3^
*CV*(*SPV*)	−0.61	< 10^−3^	−0.20	< 10^−3^
*σ*(*SPV*)	0.0004	0.99	0.53	< 10^−3^
*Sk*(*SPV*)	−0.39	< 10^−3^	−0.14	< 10^−3^

To further examine the connections between the *SPV* characteristics *θ* ∈ {*m*, *β*, *CV*(*SPV*), *σ*(*SPV*), *Sk*(*SPV*)} and *τ* ∈ {*FI*_∞_, *R*_0_} and concentrating specifically on the role of *σ*(*SPV*) and *m*, we describe two case studies below.

#### Case study 1—*σ*(*SPV*)

**Fig 6:**
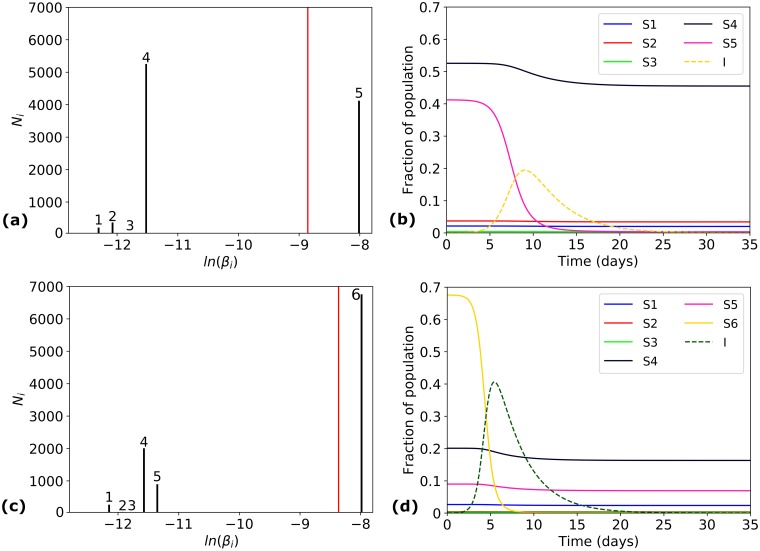
Case study 1—*σ*(*SPV*). Simulation results for epidemic pairs 1 (a)-(b) and 2 (c)-(d) in Table 3. Distribution of *β*_*i*_ values in the population (*left*): the *x*-axis represents values of *ln*(*β*_*i*_), and the *y*-axis shows values of *N*_*i*_. The red vertical line corresponds to the average susceptibility *β*. Time course of the epidemic (*right*) in terms of variables *S*_*i*_(*t*) (*solid*) for each sub-population, and *I*(*t*) (*dashed*).

Fig 5(b) shows that most of the epidemic pairs in our data set have *σ*(*SPV*) < 10^−4^*person*^−1^*day*^−1^. Although the correlation between *σ*(*SPV*) and *FI*_∞_ is not statistically significant (see Table 2), we notice that a high value of *σ*(*SPV*) (> 1.5 × 10^−4^*person*^−1^*day*^−1^) corresponds to moderate values for *FI*_∞_. We examine two (*E*, *V*) pairs with high *σ*(*SPV*); see pairs 1 and 2 in Table 3 and their corresponding epidemic dynamics in Fig 6. These two pairs have similar values for *σ*(*SPV*), and yet have significantly different epidemic sizes (0.48 for pair 1, and 0.74 for pair 2). We also see from Fig 6(b) and 6(d), that the infection runs its course faster in pair 2 than in pair 1. Both these phenomena can be explained by the fact that pair 2 has a significantly higher *β* (2.33 × 10^−4^*person*^−1^*day*^−1^, compared to 1.42 × 10^−4^*person*^−1^*day*^−1^ for pair 1). We can also see from Fig 6 that the sub-population with highest *β*_*i*_ is the one most affected by the infection, while the sub-populations with low *β*_*i*_ remain largely uninfected, in both pairs 1 and 2. We will see in further sections that *β* and *σ*(*SPV*) together, correlate well with epidemic size.

**Table 3:** Case study 1—Studying the predictive power of *σ*(*SPV*).

Pair	Ethnicity (E)	Strain (V)	*m*	*β* (×10^−4^)	*σ*(*SPV*) (×10^−4^)	*CV*(*SPV*)	*Sk*(*SPV*)	*FI*_∞_	*R*_0_
1	China North Han	A/Fukuoka-C/3/2009	5	1.42	1.59	1.12	0.36	0.48	4.27
2	China Yunnan Province Hani pop 2	A/Auckland/ 1/2009	6	2.33	1.55	0.67	−0.75	0.74	6.99

*β* and *σ*(*SPV*) have units *person*^−1^*day*^−1^. Histograms of each susceptibility profile *SPV*(*E*, *V*), together with the dynamics of each epidemic, are shown in Fig 6.

#### Case study 2—*m*

**Fig 7:**
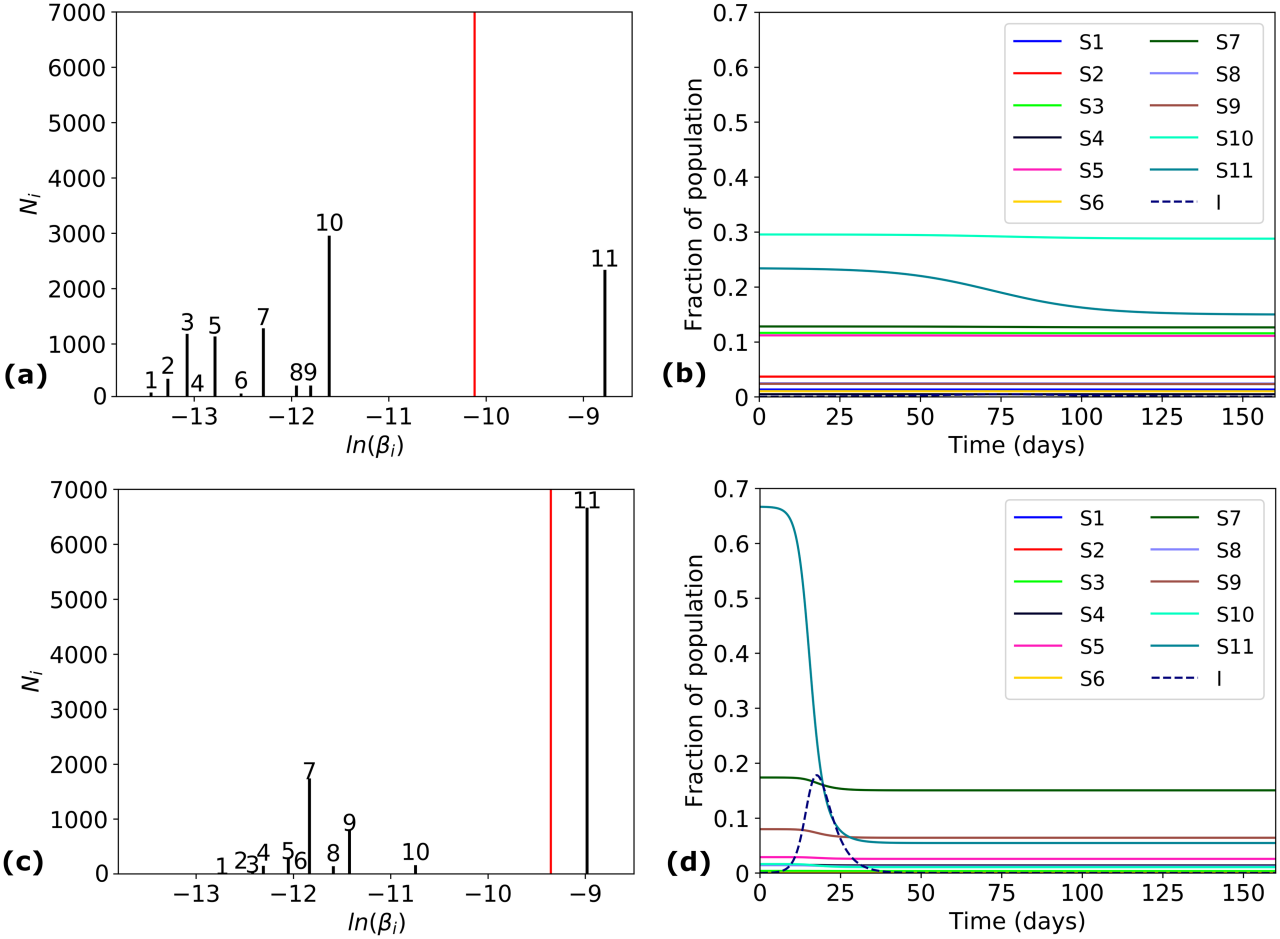
Case study 2—*m*. Simulation results for epidemic pairs 3 (a)-(b) and 4 (c)-(d) in Table 4. Distribution of *β*_*i*_ values in the population (*left*): the *x*-axis represents values of *ln*(*β*_*i*_), and the *y*-axis shows values of *N*_*i*_. The red vertical line corresponds to the average susceptibility *β*. Time course of the epidemic (*right*) in terms of variables *S*_*i*_(*t*) (*solid*) for each sub-population, and *I*(*t*) (*dashed*).

In Fig 5(d) there appears to be some negative correlation between *m* and *FI*_∞_, with larger values of *m* corresponding to smaller epidemic sizes; see Table 2. However, we note that this is more an artefact of the data than a predictive trend, and it is possible to have epidemic pairs with a large value of *m* but very different final epidemic sizes and epidemic time-course dynamics. This can be seen for example in Fig 7 for epidemic pairs 3 and 4 from Table 4. Once again, the pair with higher average susceptibility has both a larger epidemic size, and also a faster time course for the spread of the disease.

**Table 4:** Case study 2—Studying the predictive power of *m*.

Pair	Ethnicity (E)	Strain (V)	*m*	*β* (×10^−4^)	*σ*(*SPV*) (×10^−4^)	*CV*(*SPV*)	*Sk*(*SPV*)	*FI*_∞_	*R*_0_
3	Uganda Kampala pop 2	A/Canada-NFL/RV3019/ 2009	11	0.4	0.63	1.58	1.25	0.10	1.21
4	USA Alaska Yupik	A/California/ 07/2009	11	0.87	0.55	0.63	−0.71	0.66	2.60

*β* and *σ*(*SPV*) have units *person*^−1^*day*^−1^. Histograms of each susceptibility profile *SPV*(*E*, *V*), together with the dynamics of each epidemic, are shown in Fig 7.

### Dependence of *R*_0_ on *SPV* characteristics

**Fig 8:**
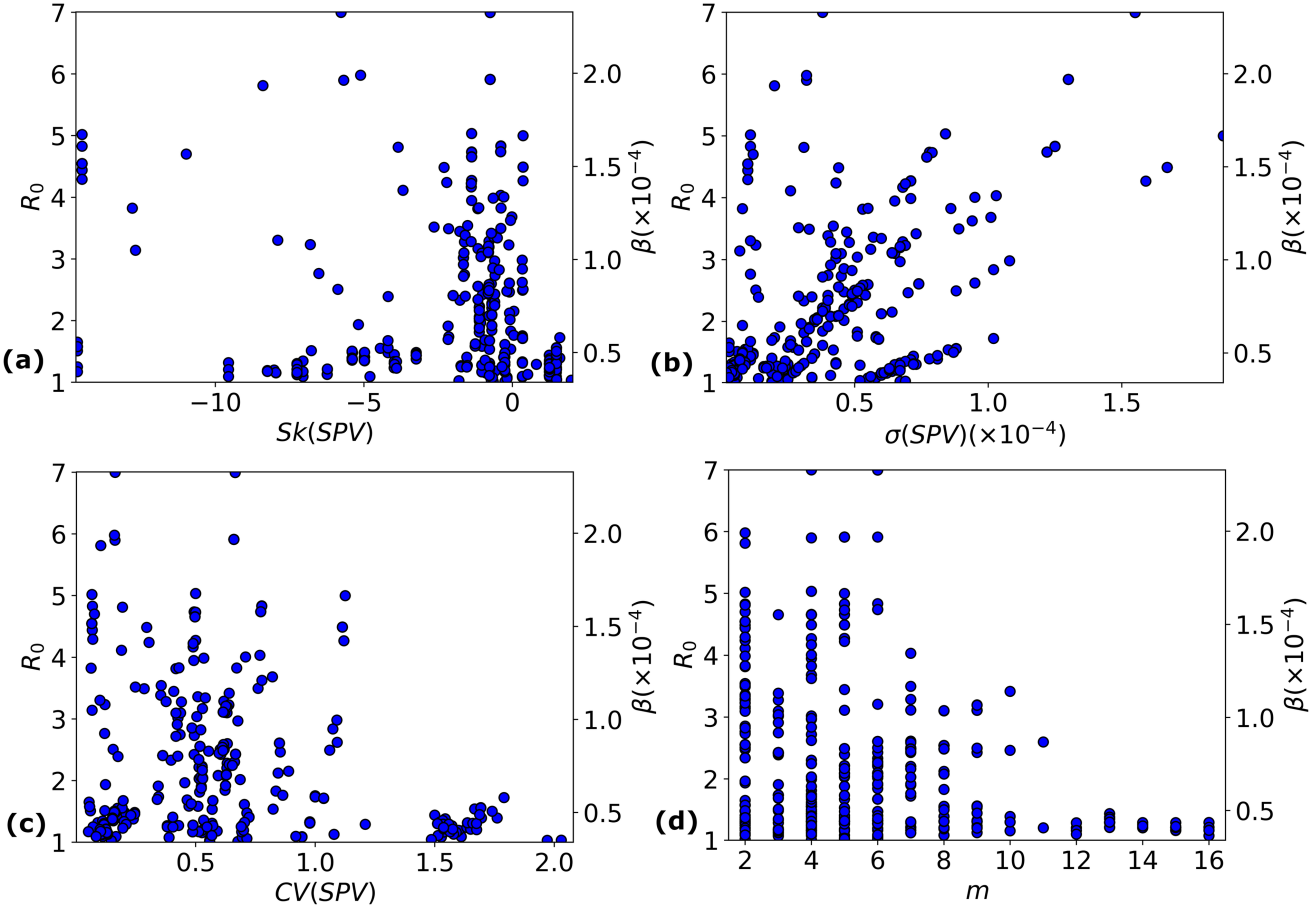
*R*_0_ as a function of *SPV* characteristics. The dependence of the basic reproduction number *R*_0_ on several characteristics of the susceptibility profile vector of each (*E*, *V*) pair considering H1N1 strains isolated in 2009: (a) skewness of the *SPV*; (b) standard deviation of the *SPV*; (c) coefficient of variation of the *SPV*; (d) number of susceptibility sub-populations, *m*. Only epidemic pairs (*E*, *V*) with 1 < *R*_0_ < 7 and *m* > 1 are plotted.

We address the question of whether *R*_0_ can be estimated from the *SPV* characteristics *θ* ∈ {*m*, *CV*(*SPV*), *σ*(*SPV*), *Sk*(*SPV*)}; see Fig 8. The linear relationship between *β* and *R*_0_ follows from Eq (16). In Fig 8(d), we once again observe that (*E*, *V*) pairs with *m* > 10 have low *R*_0_. As observed in case study 2, this is more an artefact of biases in the real data than a general trend. In Fig 8(b), we plot *σ*(*SPV*) against *R*_0_. Although *σ*(*SPV*) does not directly affect *R*_0_, we see this shape due to the relationship in the data between *β* and *σ*(*SPV*).

### Epidemic size largely correlates with select pairs of parameters

**Fig 9:**
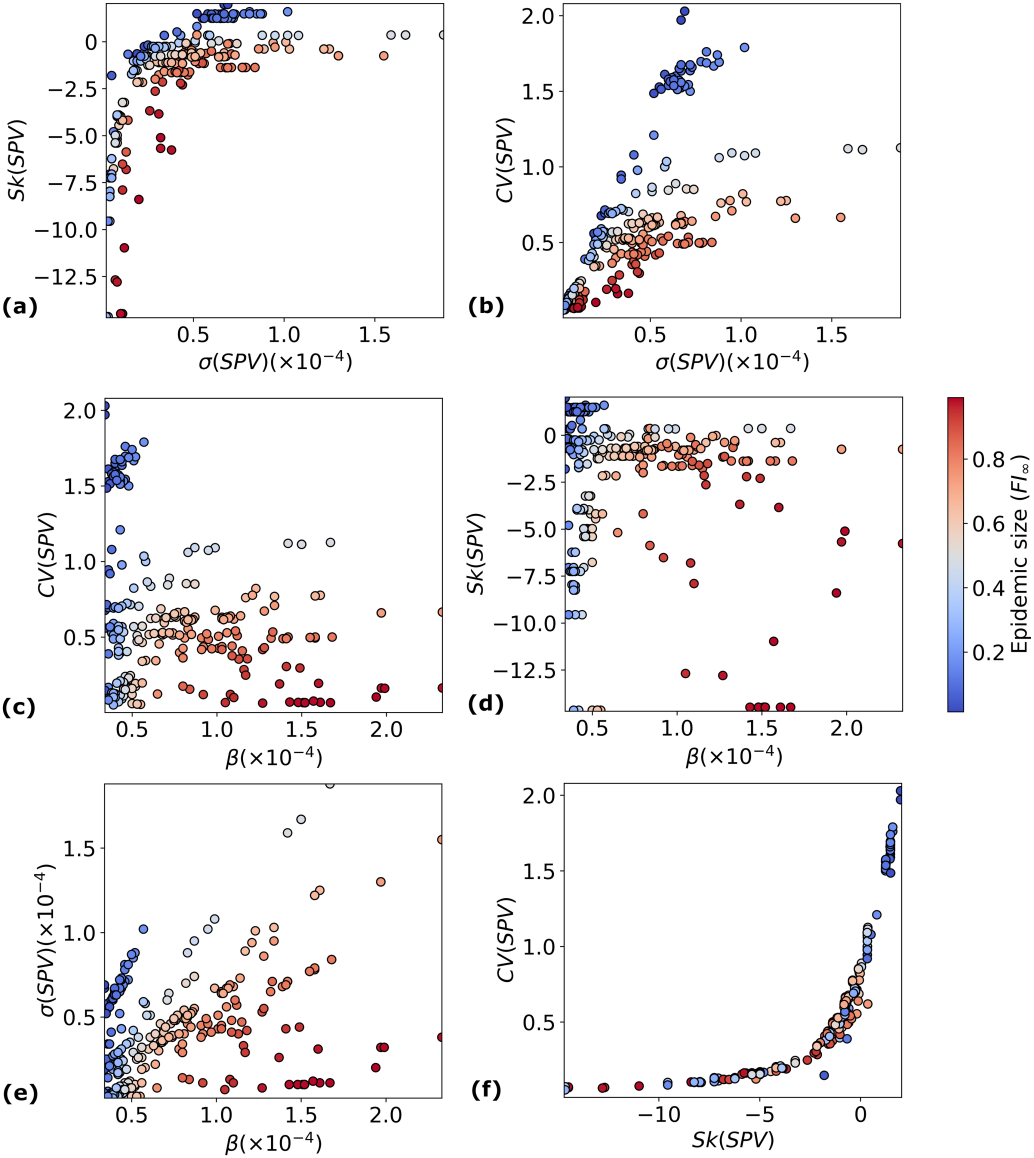
*FI*_∞_ as a function of pairs of *SPV* characteristics. (a) (*Sk*(*SPV*), *σ*(*SPV*)); (b) (*CV*(*SPV*), *σ*(*SPV*)); (c) (*CV*(*SPV*), *β*); (d) (*Sk*(*SPV*), *β*); (e) (*σ*(*SPV*), *β*); (f) (*CV*(*SPV*), *Sk*(*SPV*)). *FI*_∞_ is shown as a colourbar.

Earlier, we examined the correlation between *FI*_∞_ and the *SPV* characteristics *θ* ∈ {*m*, *β*, *CV*(*SPV*), *σ*(*SPV*), *Sk*(*SPV*)} independently. This raises the question of whether accounting for pairs of such parameters might provide a more accurate prediction of *FI*_∞_. We study here how pairs of parameters are related to epidemic size in all (*E*, *V*) pairs with 1 < *R*_0_ < 7 and *m* > 1. We find that pairs involving the average susceptibility *β*, as well as the heterogeneity parameters *Sk*(*SPV*), *CV*(*SPV*) and *σ*(*SPV*) are better predictors of the final epidemic size than these quantities individually. Plots involving these parameters are shown in Fig 9, while multiple correlation coefficients are shown in Table 5. All other parameter pairs are plotted in supporting information; see S2 Fig. In particular, note that:

- Epidemic pairs containing a susceptibility profile vector leading to large values of *CV*(*SPV*), small values of *β*, and positive *Sk*(*SPV*) experience smaller final epidemic sizes.
- Epidemic pairs with positive *Sk*(*SPV*) are also the ones with small average susceptibility, and they lead to small final epidemic sizes.

**Table 5:** Correlation coefficients *r*((*θ*_1_, *θ*_2_), *τ*) between summary statistics of the epidemic and *SPV* characteristics pairs.

*SPV* Characteristic Pair (*θ*_1_, *θ*_2_)	*FI*_∞_	*R*_0_
(*β*, *m*)	0.81	1.0
(*β*, *CV*(*SPV*))	0.88	1.0
(*β*, *σ*(*SPV*))	0.87	1.0
(*β*, *Sk*(*SPV*))	0.80	1.0
(*m*, *CV*(*SPV*))	0.62	0.24
(*m*, *σ*(*SPV*))	0.55	0.72
(*m*, *Sk*(*SPV*))	0.53	0.24
(*CV*(*SPV*), *σ*(*SPV*))	0.78	0.86
(*CV*(*SPV*), *Sk*(*SPV*))	0.62	0.20
(*σ*(*SPV*), *Sk*(*SPV*))	0.48	0.75

From Fig 9, we see that for a given *β*, *FI*_∞_ decreases with increasing *σ*(*SPV*). It also decreases as *Sk*(*SPV*) is made more positive, or as *CV*(*SPV*) is increased. This shows that for intermediate values of *β* such as the ones shown in Fig 9, a higher spread in *β*_*i*_ values helps to protect the population against the epidemic spread. In other words, a population with higher genetic heterogeneity in susceptibility to a virus, leading to susceptibility sub-populations with a large spread in susceptibilities, can be expected to have a smaller epidemic than a population where most of the people have similar susceptibility, despite both populations being non-homogeneous in susceptibility.

We also observe that for a given value of *β*, an (*E*, *V*) pair with *Sk*(*SPV*) > 0 or only slightly negative corresponds to a smaller *FI*_∞_ than one for which *Sk*(*SPV*) is a large negative value. We interpret this in the following way: populations containing a small sub-set of individuals with heightened susceptibility, but in which most of the individuals are less susceptible, are better protected against the disease than populations where the susceptibility is more uniformly distributed, even if the mean susceptibility is the same.

We provide in Table 5 multiple correlation coefficients

$$r(\left(\theta_{1},\theta_{2}\right),\tau )=\frac{\sqrt{r{\left(\theta_{1},\tau \right)}^{2}+r{\left(\theta_{2},\tau \right)}^{2}-2r\left(\theta_{1},\tau \right)r\left(\theta_{2},\tau \right)r\left(\theta_{1},\theta_{2}\right)}}{\sqrt{1-r{\left(\theta_{1},\theta_{2}\right)}^{2}}}\in \left(0,1\right)$$

between our summary statistics *τ* ∈ {*FI*_∞_, *R*_0_} and *SPV* characteristics pairs (*θ*_1_, *θ*_2_) ∈ {*m*, *β*, *CV*(*SPV*), *σ*(*SPV*), *Sk*(*SPV*)}^2^.

### Predictions broadly track trends in pH1N1 (2009) burden

The 2009 pandemic of H1N1 was closely tracked by many organisations in the world, including the World Health Organization (WHO). For example, [62, Fig 3] indicates that certain areas of the world experienced a larger number of cases than others. In particular, we see that China and Japan experienced worse epidemics than Russia, which tends to have relatively smaller epidemics.

**Fig 10:**
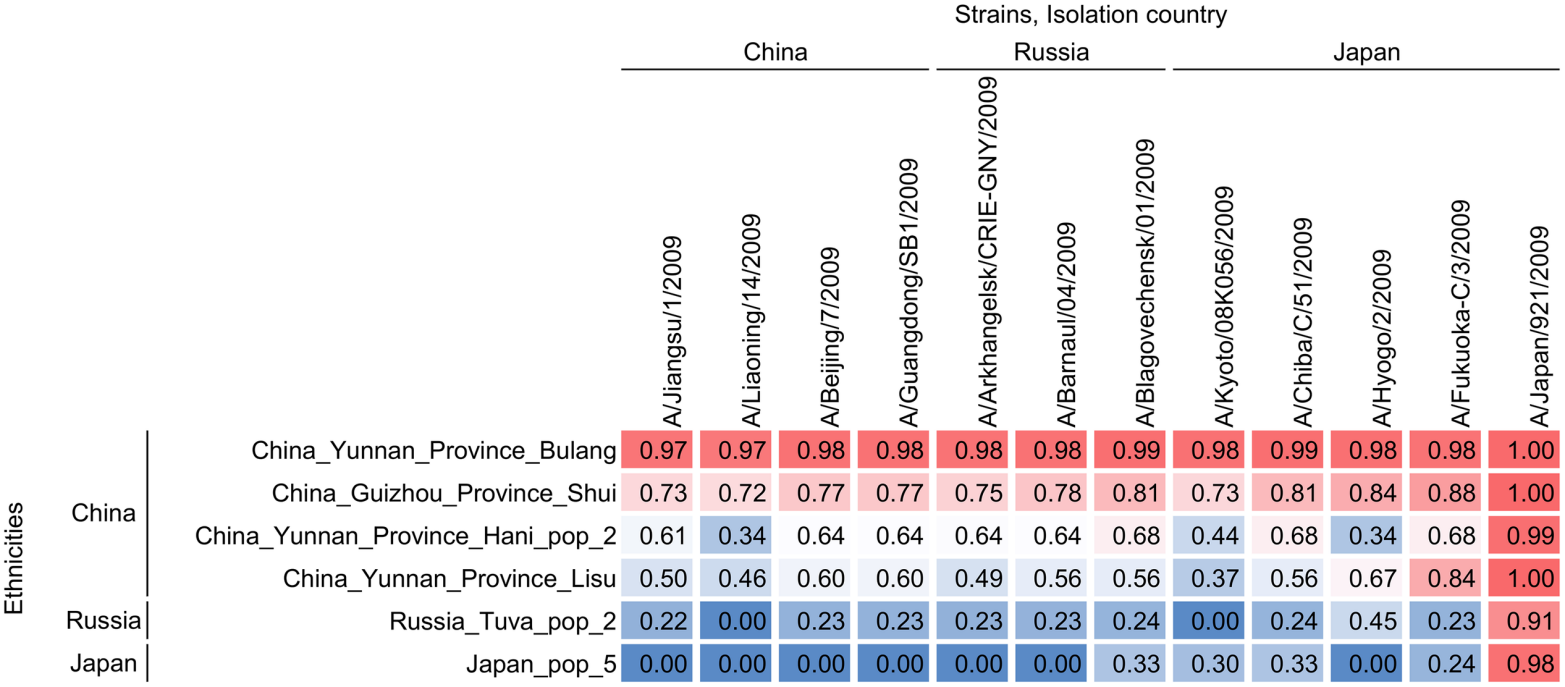
Qualitative trends captured by our model. Epidemic sizes predicted by our model for different ethnicities and strains corresponding to China, Russia, and Japan, during the 2009 influenza A/H1N1 pandemic. Qualitatively, the predictions broadly track trends observed worldwide.

To compare the predictions of our model with these observations, we select viral strains isolated in these regions during the 2009 pandemic, and ethnicities corresponding to these countries. We would like to mention here that our model works with individual ethnicities, while the data available is for countries, which are comprised of multiple ethnicities. We find that different ethnicities from the same country experience widely differing epidemic sizes for the same viral strain; see S1 File. For this comparison, we select ethnicities available in our data set from each of these countries, for which the predictions most closely resemble the observations in [62, Fig 3]; see Fig 10. As can be seen in Fig 10, our method predicts that most Chinese ethnicities will experience severe epidemics regardless of the viral strain. On the other hand, Russia and Japan are predicted to experience smaller epidemics for most viral strains. However, we note that for most of the Japanese strains, the Japanese ethnicity will suffer larger epidemic sizes than the Russian one, thus qualitatively agreeing with what can be observed in [62, Fig 3].

An interesting case is the strain A/Japan/921/2009 (H1N1), which was one of the strains circulating in Japan during the 2009 pandemic. This strain is predicted to cause severe epidemics in most ethnicities, and this holds true across all the 61 ethnicities considered in the data set.

The ethnicity Russia Tuva Pop 2 is predicted to experience moderate epidemics for viral strains isolated in Russia, but a slightly worse epidemic for the strain A/Hyogo/2/2009 (H1N1), isolated in Japan. Thus, our methods bear out the idea that the severity of an influenza epidemic in a given country should not be dictated entirely by the genetic makeup of the hosts, but should also depend on the particular strain of the pathogen circulating in this country. Our predictions suggest that the ability of HLA class-I alleles in the ethnicity Russia Tuva pop 2 to present epitopes from the influenza A (H1N1) virus changes significantly across different viral strains.

The results described above show that even with a model that only incorporates susceptibility heterogeneities in terms of epitope presentation through HLA class-I alleles, we can qualitatively explain some essential trends observed across the world during the 2009 H1N1 pandemic. This serves as a qualitative validation of our methodology. Moreover, our results suggest that while some trends in influenza spread worldwide can be explained by the average susceptibility of each ethnicity to each strain, others might have an explanation related to the particular genetic diversity within each ethnicity for a given strain. For example, when analysing pairs 5 and 6 in Table 6, we can see that the same value of *β* can lead to different epidemic sizes for the same strain when considering the China Yunnan Province Lisu and the Japan pop 5 ethnicities. This is likely related to the fact that *Sk*(*SPV*) is significantly more negative for the Chinese ethnicity, and the coefficient of variation is smaller, leading to a larger epidemic size. A similar behaviour can be seen when considering pairs 7-9 in Table 6. Larger reproduction numbers can still arise from smaller epidemic sizes if *Sk*(*SPV*) is closer to 0 (or positive), and for more heterogeneous populations (larger values of *CV*(*SPV*)), which might explain smaller epidemic sizes in, for example, the Kenya Luo ethnicity compared to the Chinese ones [62, Fig 3].

**Table 6:** Select case studies to study the observed behaviour in Fig 10.

Pair	Ethnicity (E)	Strain (V)	*m*	*β*(×10^−4^)	*σ*(*SPV*) (×10^−4^)	*CV*(*SPV*)	*Sk*(*SPV*)	*FI*_∞_	*R*_0_
5	China Yunnan Province Lisu	A/Kyoto/ 08K056/2009	2	0.42	0.03	0.07	−14.64	0.37	1.25
6	Japan pop 5	A/Kyoto/ 08K056/2009	4	0.42	0.24	0.57	−0.77	0.30	1.27
7	China Guizhou Province Shui	A/Fukuoka-C/3/2009	2	0.84	0.13	0.15	−5.87	0.88	2.51
8	China Yunnan Province Lisu	A/Fukuoka-C/3/2009	5	1.15	0.47	0.41	−1.77	0.84	3.45
9	Kenya Luo	A/Japan/ 921/2009	4	1.23	1.01	0.82	−0.02	0.67	3.68

Epidemic pairs with similar *R*_0_ have different epidemic sizes, governed by their genetic heterogeneity. *β* and *σ*(*SPV*) have units *person*^−1^*day*^−1^.

### Similar trends are observed in H1N1 strains isolated in years other than 2009

We carry out parameter estimation and simulations as described in previous sections, for 85 strains of H1N1 influenza isolated in years other than 2009. This includes 15 strains isolated before 2000, 21 strains isolated between 2000 and 2008 (inclusive), and 49 strains isolated after 2009. We find that the trends identified in Fig 9 apply even for these strains; see supporting information S3 Fig.

### Response of some indigenous ethnicities to H1N1

Several studies have reported that during the 2009 pandemic, indigenous ethnicities experienced more severe epidemics than their non-indigenous counterparts [63–65]. The indigenous ethnicities in our data set are USA Alaska Yupik, Australia Yuendumu Aborigine, and Australia Cape York Peninsula Aborigine. We find that the ethnicity USA Alaska Yupik is always predicted to have a worse epidemic than non-indigenous ethnicities from the USA, irrespective of the strain being considered. Since our data set does not include any non-indigenous ethnicities from Australia, we are unable to verify whether or not a similar statement holds true for the Australian aboriginal ethnicities.

In general, we find the ethnicity Australia Cape York Aborigine, with average *FI*_∞_ = 0.14 when considering all 166 viral strains, is predicted to experience a marginally worse epidemic than Australia Yuendumu Aborigine whose average *FI*_∞_ = 0.08. Interestingly, this trend is reversed when we focus on the strains A/Auckland/1/2009 and A/Auckland/597/2000 isolated in Australia. For these strains, Australia Cape York Aborigine has *R*_0_ < 1 for both these strains, but Australia Yuendumu Aborigine has *R*_0_ = 1.49 for the strain A/Auckland/1/2009; see Table 7.

**Table 7:** Case study 3—Studying Australian aboriginal ethnicities.

Pair	Ethnicity (E)	Strain (V)	*m*	*β* (×10^−4^)	*σ*(*SPV*) (×10^−4^)	*CV*(*SPV*)	*Sk*(*SPV*)	*FI*_∞_	*R*_0_
10	Australia Yuendumu Aborigine	A/Auckland /1/2009	1	0.5	0	0	*NA*	0.57	1.49
11	Australia Yuendumu Aborigine	A/Auckland /597/2000	1	0.28	0	0	*NA*	0.0006	0.82
12	Australia Cape York Peninsula Aborigine	A/Auckland /1/2009	3	0.33	0.1	0.3	−1.8	0.006	0.98
13	Australia Cape York Peninsula Aborigine	A/Auckland /597/2000	3	0.19	0.05	0.26	−1.93	0.0002	0.58

*β* and *σ*(*SPV*) have units *person*^−1^*day*^−1^. Since *Sk*(*SPV*) can only be calculated when there are *m* > 1 sub-populations, *Sk*(*SPV*) = *NA* (Not Applicable) for epidemic pairs 10 and 11.

Based on the observations during the 2009 pandemic, it has been suggested that aboriginal communities should be prioritised during vaccination [63, 64]. However the predictions in Table 7 suggest that at least from the perspective of HLA alleles and downstream CTL response, each influenza strain and each aboriginal community needs to be assessed independently. Using our model, it is possible to predict whether or not a new strain will cause a worse epidemic than a strain in the data set, within the constraints of the assumptions made. Predictions such as these could help optimise the deployment of resources when combating a new strain of influenza.

### High risk alleles for one strain do not always correlate with severe epidemics in general

The frequency of the HLA class-I allele HLA-A*24 has been found to correlate with mortality rate due to the pandemic H1N1 (2009) influenza virus [36]. We rank ethnicities in our data set in descending order of their average *FI*_∞_ across all 166 strains of influenza, and find that the ethnicity USA Alaska Yupik has the highest prevalence of allele HLA-A*24:02, and also has the worst average epidemic size; see Table 8. The ethnicity with the next highest frequency of allele HLA-A*24:02, Japan Central, has very low average epidemic size, and ranks 52^*nd*^ among 61 ethnicities. The ethnicity Japan pop 3 has comparable frequency of the allele HLA-A*24:02 as Japan Central, but is ranked 28^*th*^ based on its average epidemic size. These results show that an allele whose frequent occurrence correlates with a high risk for one influenza strain, does not always correlate with a severe epidemic when considering influenza strains in general. Rather, we need to estimate the full profile of the *SPV*, or at least the summary characteristics with strong correlation as described in previous sections.

**Table 8:** Top 3 ethnicities with high risk allele HLA-A*24:02.

Ethnicity (E)	Allele frequency rank	Frequency	Average *FI*_∞_	Average *FI*_∞_ rank
USA Alaska Yupik	1	58%	0.68	3
Japan Central	2	38%	0.009	52
Japan pop 3	3	36%	0.02	28

Higher average *FI*_∞_ rank implies more severe predicted epidemic. Ranks are out of the 61 ethnicities considered in this data set.

### Synthetic data supports the observed behaviour

Does the behaviour discussed in the preceding sections rely on correlations between *SPV* characteristics that are specific to the epidemic pairs we analyse? These correlations arise directly from genetic heterogeneities at the HLA genotype level corresponding to the 61 ethnicities and 166 viral strains considered here. However, we could frame our questions more generally. For example, we could ask if a positive skewness of the *SPV* would always be a protective characteristic for the population, given a fixed average *β*?

To address these and similar questions, we construct a synthetic data set of 10^4^ epidemic pairs created within the following parameter ranges:

$$\begin{array}{ccc} m∼U_{int}\left(\left\{2,\ldots ,15\right\}\right), & & \\ e_{i}=2\times u\times 10^{p_{i}}, & & 1\leq i\leq m,\\ u∼U\left(0,1\right),\;p_{i}∼U\left(log_{10}\left(e_{min}\right),log_{10}\left(e_{max}\right)\right), & & 1\leq i\leq m,\\ N_{i}∼U_{int}\left(\left\{1,\ldots ,N\right\}\right), & & 1\leq i\leq m\;s.t.\;\sum_{i=1}^{m}N_{i}=N, \end{array}$$

where *e*_*min*_ and *e*_*max*_ are the minimum and maximum values of *e*_*i*_ in the real data set analysed in previous sections. These distributions have been chosen so that we obtain 10^4^ epidemic pairs with values in the interval 1 < *R*_0_ < 7, *m* > 1, with *N*_*i*_ and *β*_*i*_ distributed within ranges that are comparable to those of the original data set.

**Fig 11:**
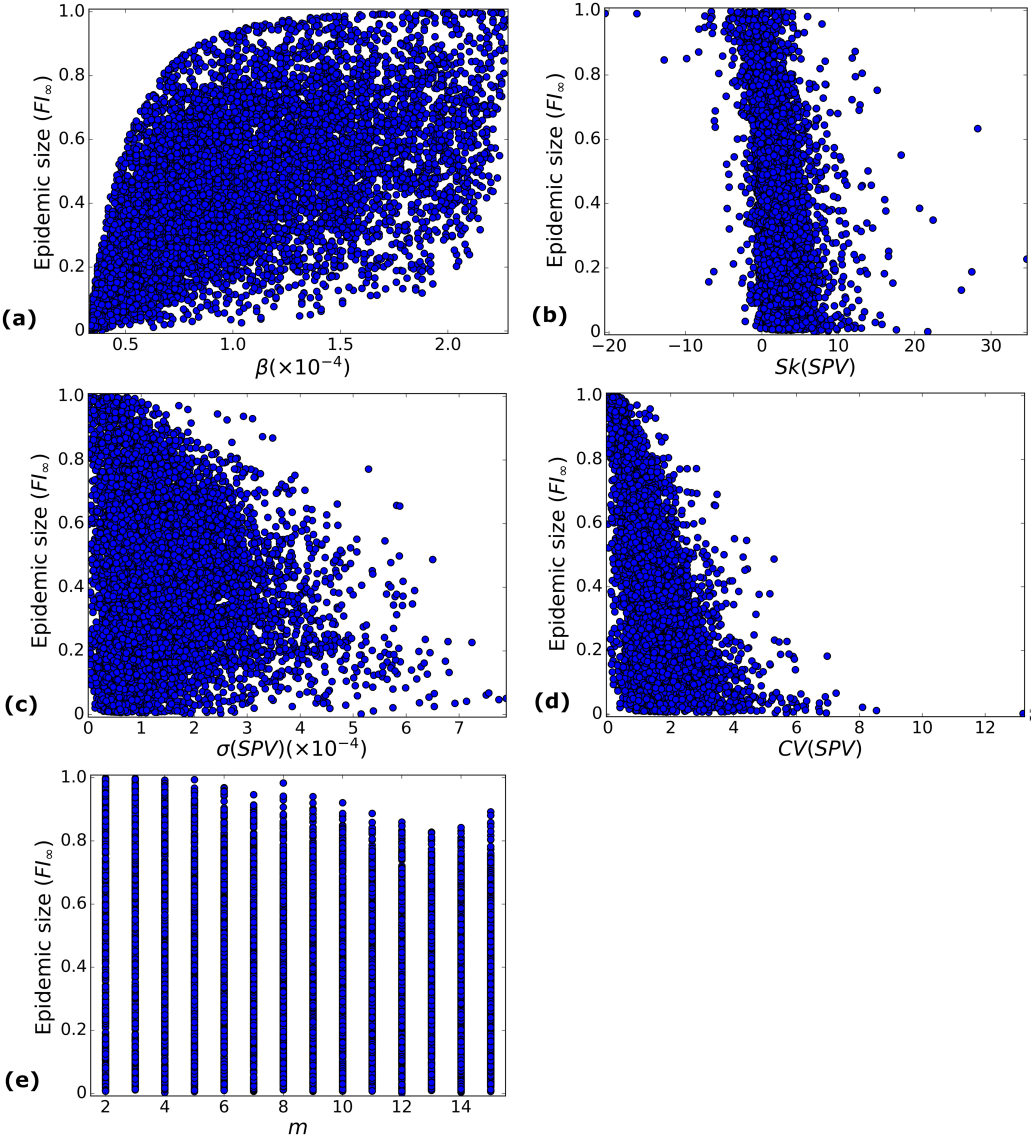
*FI*_∞_ as a function of *SPV* characteristics—Synthetic data set. Dependence of epidemic size on several characteristics of the *SPV* is analysed for the synthetic data set described in the text. (a) *β*; (b) *Sk*(*SPV*); (c) *σ*(*SPV*); (d) *CV*(*SPV*); (e) *m*.

For this synthetic data set, we plot in Fig 11 the predicted final epidemic size as a function of the different *SPV* characteristics. In Tables 9 and 10, correlation coefficients for single and paired *SPV* characteristics, and summary statistics *FI*_∞_ and *R*_0_, are provided for the epidemic pairs in this synthetic data set. A direct inspection of results in Fig 11 and Tables 9 and 10 lead to the following conclusions:

- Large values of *β* lead to larger epidemic sizes. However, *β* alone can not explain *FI*_∞_, and other characteristics of the *SPV* need to be taken into account, as for the original data set; see Fig 11(a).
- Positive skewness leads to smaller epidemic sizes than negative skewness scenarios, as observed for the original data set; see Fig 11(b).
- The larger the heterogeneity (in terms of *σ*(*SPV*) or *CV*(*SPV*)), the more protected the population is against epidemic spread. This is not a consequence of the value of *m*. Rather, it is the particular combination of *β*_*i*_ and *N*_*i*_ values which has an impact on the epidemic dynamics; see Fig 11(c)–11(e).

**Table 9:** Correlation coefficients *r*(*θ*, *τ*) between summary statistics of the epidemic and *SPV* characteristics for the synthetic data set.

*SPV* Characteristic *θ*	*FI*_∞_	p-value	*R*_0_	p-value
*m*	−0.2	< 10^−3^	0.02	0.03
*β*	0.62	< 10^−3^	1.00	< 10^−3^
*CV*(*SPV*)	−0.58	< 10^−3^	−0.06	< 10^−3^
*σ*(*SPV*)	−0.06	< 10^−3^	0.61	< 10^−3^
*Sk*(*SPV*)	−0.40	< 10^−3^	−0.12	< 10^−3^

**Table 10:** Correlation coefficients *r*((*θ*_1_, *θ*_2_), *τ*) between summary statistics of the epidemic and *SPV* characteristics pairs, for the synthetic data set.

*SPV* Characteristic Pair (*θ*_1_, *θ*_2_)	*FI*_∞_	*R*_0_
(*β*, *m*)	0.66	1.0
(*β*, *σ*(*SPV*))	0.83	1.0
(*β*, *Sk*(*SPV*))	0.70	1.0
(*β*, *CV*(*SPV*))	0.83	1.0
(*m*, *σ*(*SPV*))	0.21	0.62
(*m*, *Sk*(*SPV*))	0.34	0.14
(*m*, *CV*(*SPV*))	0.58	0.07
(*CV*(*SPV*), *σ*(*SPV*))	0.73	0.88
(*CV*(*SPV*), *Sk*(*SPV*))	0.58	0.13
(*σ*(*SPV*), *Sk*(*SPV*))	0.42	0.75

## Discussion

Theoretical studies on epidemiological spread of disease in the presence of susceptibility heterogeneities have shown that final epidemic size is typically lower when susceptibility sub-populations are factored in, as compared to the case of homogeneous susceptibility [21, 44, 61]. We find that this result holds true when the sub-population sizes and disease transmission rates are informed by real-world data about immunological factors. The novelty in our approach is to propose how the susceptibility profile vector can be estimated from genetic sequence data, so that we can then deal with particular *SPVs* that might exist in reality for different ethnicities and viral strains. We also show that some summary statistics of the *SPV* (such as the skewness or the coefficient of variation) can help to better understand the predicted final size of the epidemic.

A limitation of our model is that factors such as age, prior infection history and vaccination are not included. While there have been studies which collect and analyse such data for small cohorts [47, 48, 65], gathering such information on the global scale required for this analysis requires the formation of consortia such as those existing for diseases such as cancer [37]. Also, we make the strong simplifying assumption that all aspects of the innate and adaptive immune system not affected by HLA class-I presentation can be pooled into a single proportionality constant, and are considered uniform among individuals within an ethnicity, and across ethnicities. While this helped focus the analysis on the role of HLA alleles in disease spread, incorporating other aspects of the immune system into epidemiological models is an important problem that must be addressed. Due to these limitations, predictions made by our model can only be used to draw *comparisons* between different epidemic pairs, particularly epidemic pairs consisting of the same ethnicity and different viral strains, and not for making absolute quantitative predictions.

A number of extensions of the line of work presented in this manuscript are possible. Presentation of epitopes by HLA class-I alleles is preceded by a number of steps including internalisation of the virus, proteasomal cleavage of viral proteins into shorter peptides, and transport of peptides through the TAP transport system [31]. The epitope prediction tools used in this work do not explicitly consider all these pre-processing steps in any single tool. Also, the prediction algorithms have lower accuracy for rare alleles. The model can be improved by plugging in different epitope prediction methods which overcome these limitations. Also, it would be useful to establish a more accurate, quantitative connection between *s*_*i*_ and *e*_*i*_ than the simple inverse relation we have assumed. Two other possible mathematical forms, *s*_*i*_ ∝ 1/*ln*(*e*_*i*_ + 1) and *s*_*i*_ ∝ 1/(*e*_*i*_ + 1)^2^, are explored in the supporting information; see S4 Fig.

Spatial heterogeneities are known to allow for disease persistence, since asynchrony in the epidemic spread among different sub-populations located in different geographical locations can allow for global persistence, even if the epidemic locally dies out [19]. Since HLA alleles are inherited, it can be expected that families and households will have similar HLA genotypes, potentially introducing spatial inhomogeneity in the distribution of HLA alleles in a population. If such spatial information regarding HLA genotypes were gathered, it would be interesting to study how this affects epidemic dynamics and persistence. An agent based model incorporating variations in agent susceptibility along the lines indicated here, along with spatial information regarding each susceptible agent, would provide an idea of how such factors might modify the general conclusions described in this paper. A network model incorporating the social structure of individual contacts would indicate if the combination of varied susceptibility with a specified contact network structure between individuals might accelerate epidemic progress or retard it.

### Conclusions

The incorporation of within-host immunological information into population-level epidemic models is a major challenge for epidemiological modeling [30]. In this paper, we address this question in a specific case, by modeling the impact of genetic diversity in terms of the HLA class-I genotype on the predicted epidemic dynamics of H1N1 influenza. To do this, we made use of HLA allele frequencies measured across different ethnicities, focusing on the number of high affinity epitopes presented by individuals within 61 ethnicities and for 81 H1N1 influenza A viral strains isolated in 2009 as well as 85 H1N1 influenza A viral strains isolated in other years. Our main hypothesis was that the susceptibility of individuals in a given ethnicity, for a given viral strain, is inversely proportional to the number of high affinity epitopes that these individuals can present. We then used a multi-compartment SIR model to study the spread dynamics of influenza for each (ethnicity, viral strain) epidemic pair, where the final epidemic size *FI*_∞_ and the basic reproduction number *R*_0_ are used as the summary statistics for the purpose of comparison.

While the average susceptibility *β* is a central parameter, the susceptibility profile corresponding to each epidemic pair also plays an important role governing epidemic spread. In particular, when analysing epidemics with intermediate values of *β* (*i.e.*, intermediate values of *R*_0_), more heterogeneous susceptibility profiles, as well as profiles showing positive skewness *Sk*(*SPV*), are more protective for the population as a whole against H1N1 influenza. Our model only considers heterogeneity from the perspective of the ability of a person’s HLA genotype to present epitopes from a given virus. However, even if at a qualitative level, our results support the idea that having a wide variety of HLA alleles represented among its individuals, resulting in a wide range of susceptibilities, benefits a population as a whole in terms of restricting the spread of an infectious disease.

Although our model does not incorporate other factors such as social and economic characteristics of each particular population or potential different infectivities for each viral strain, our results qualitatively capture several central trends of influenza spread worldwide. Thus, we can conclude that susceptibility of individuals in terms of the HLA genotype is an important factor that could explain the spread potential of different influenza viral strains among different ethnicities and populations. While some of these trends can just be explained due to larger or smaller values of *R*_0_ (*i.e.*, the average susceptibility *β*), the reason for small epidemic sizes occurring for some particular ethnicities and viral strains might be related to the existence of high genetic diversity resulting in a wide range of susceptibilities in these populations, for these viral strains, with a positively skewed susceptibility profile vector.

## Supporting information

S1 Fig Variations in *SPV* characteristics, non-2009 strains. Histograms for the values of the different susceptibility profile vector characteristics for the 5, 185 epidemic pairs involving H1N1 strains isolated in years other than 2009: (a) *σ*(*SPV*); (b) *Sk*(*SPV*); (c) *CV*(*SPV*); (d) *m*; and (e) *β*.
(TIF)

S2 Fig *FI*_∞_ as a function of other pairs of *SPV* characteristics, 2009 strains. (a) (*CV*(*SPV*), *m*); (b) (*Sk*(*SPV*), *m*); (c) (*σ*(*SPV*), *m*) and (d) (*β*, *m*).
(TIF)

S3 Fig *FI*_∞_ trends hold for H1N1 strains isolated before and after 2009. *FI*_∞_ as a function of pairs of *SPV* characteristics. (a) (*Sk*(*SPV*), *σ*(*SPV*)); (b) (*CV*(*SPV*), *σ*(*SPV*)); (c) (*CV*(*SPV*), *β*); (d) (*Sk*(*SPV*), *β*); (e) (*σ*(*SPV*), *β*); (f) (*CV*(*SPV*), *Sk*(*SPV*)). *FI*_∞_ is shown as a colourbar.
(TIF)

S4 FigOther mathematical forms for $s_{i}\propto \frac{1}{e_{i}}$.The results presented in the paper use the form $s_{i}\propto \frac{1}{e_{i}}$ (column 1). Two other mathematical forms, $s_{i}\propto \frac{1}{ln(e_{i}+1)}$ (column 2) and $s_{i}\propto \frac{1}{{\left(e_{i}+1\right)}^{2}}$ (column 3) are explored here, for all 61 ethnicities and 166 viral strains. Only the pairs of *SPV* characteristics found to have high correlation with *FI*_∞_ are shown. (a, b, c) (*σ*(*SPV*), *β*); (d, e, f) (*CV*(*SPV*), *β*); (g, h, i) (*Sk*(*SPV*), *β*). *FI*_∞_ is shown as a colourbar. Trends in epidemic size hold across all considered mathematical forms.(TIF)

S1 File All calculated parameters. Parameters *m*, *β*, *β*_*i*_, *x*_*i*_, *σ*(*SPV*), *CV*(*SPV*), *Sk*(*SPV*), *FI*_∞_ and *R*_0_ for all epidemic pairs in the data set.
(XLSX)
