# Supplementary figures and images for: Role of genetic heterogeneity in determining the epidemiological severity of H1N1 influenza
Source: PLoS Comput Biol. 2018 Mar 21;14(3):e1006069. doi: 10.1371/journal.pcbi.1006069 (PMC5880410; doi:10.1371/journal.pcbi.1006069)

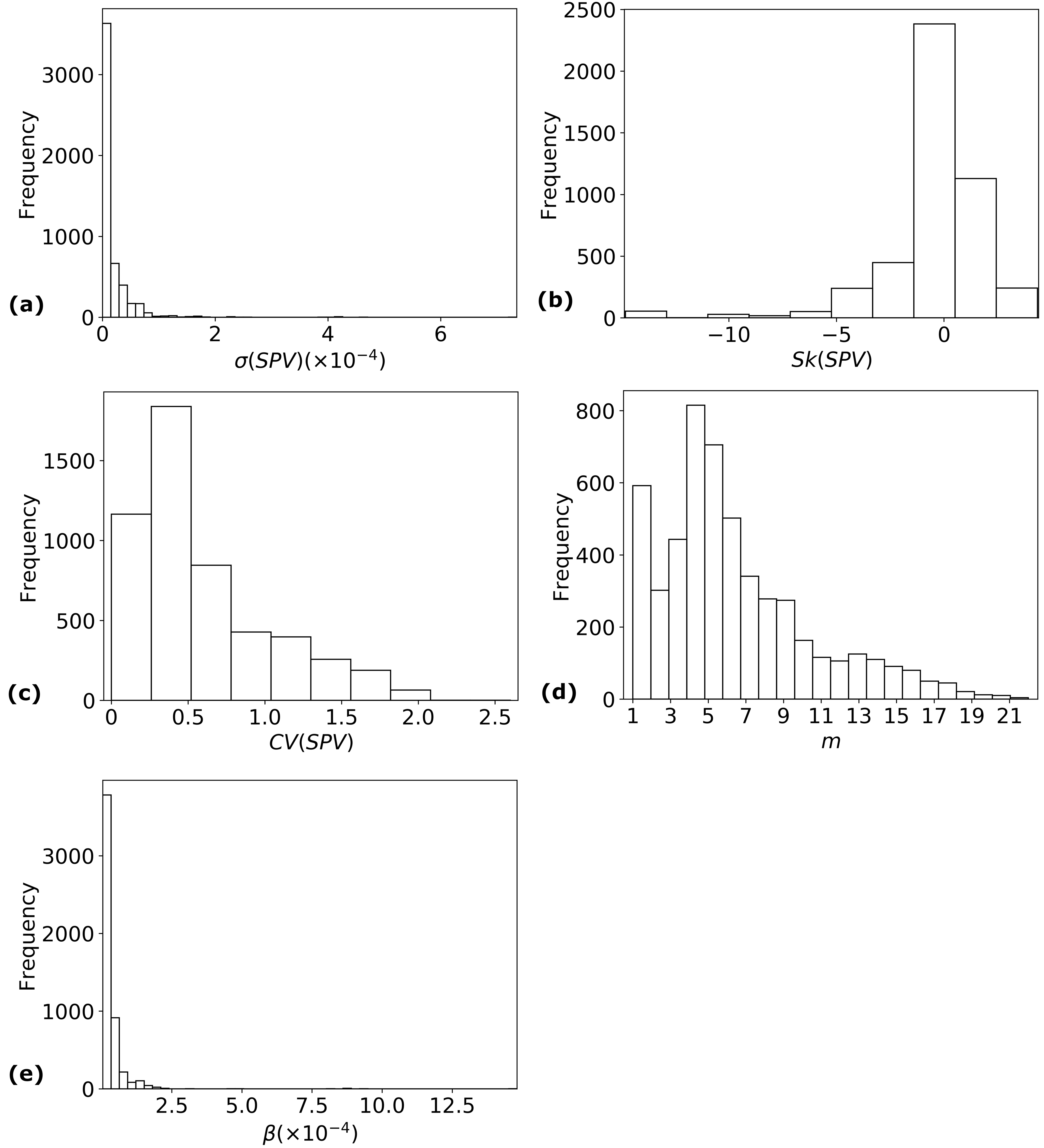

Supplement: S1 Fig — Histograms for the values of the different susceptibility profile vector characteristics for the 5, 185 epidemic pairs involving H1N1 strains isolated in years other than 2009: (a) σ(SPV); (b) Sk(SPV); (c) CV(SPV); (d) m; and (e) β. (TIF) [file pcbi.1006069.s001.tif]

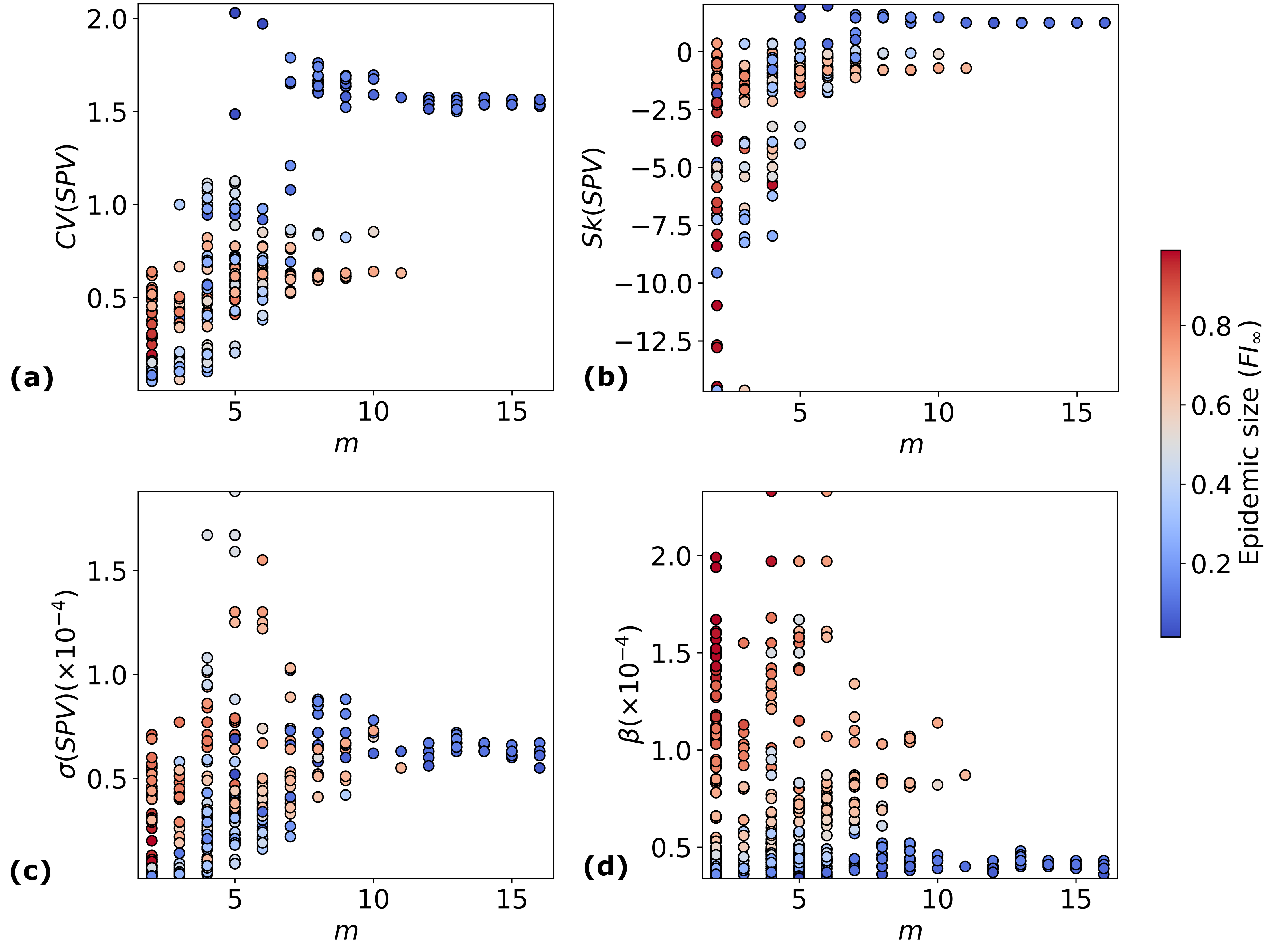

Supplement: S2 Fig — (a) (CV(SPV), m); (b) (Sk(SPV), m); (c) (σ(SPV), m) and (d) (β, m). (TIF) [file pcbi.1006069.s002.tif]

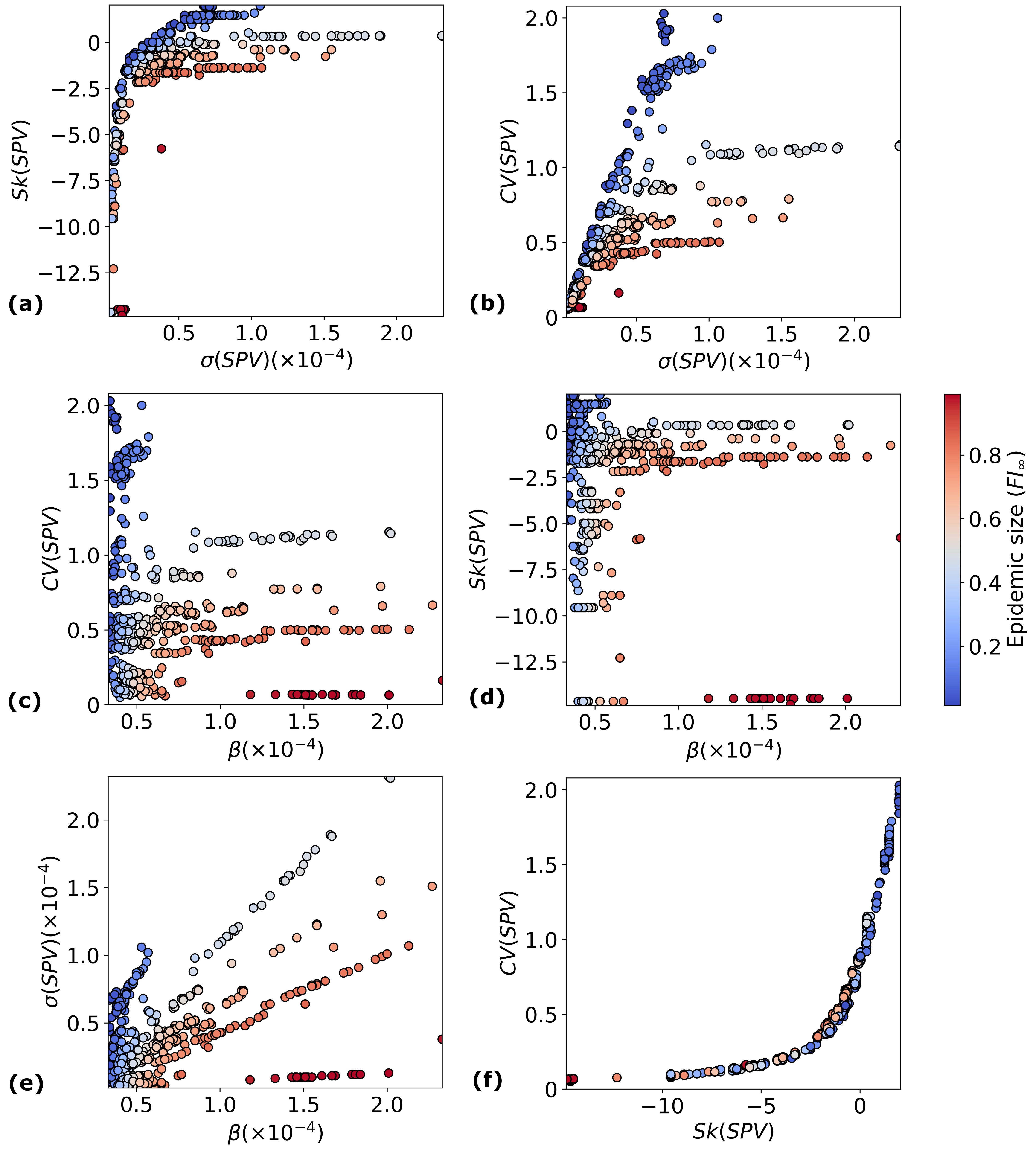

Supplement: S3 Fig — FI∞ as a function of pairs of SPV characteristics. (a) (Sk(SPV), σ(SPV)); (b) (CV(SPV), σ(SPV)); (c) (CV(SPV), β); (d) (Sk(SPV), β); (e) (σ(SPV), β); (f) (CV(SPV), Sk(SPV)). FI∞ is shown as a colourbar. (TIF) [file pcbi.1006069.s003.tif]

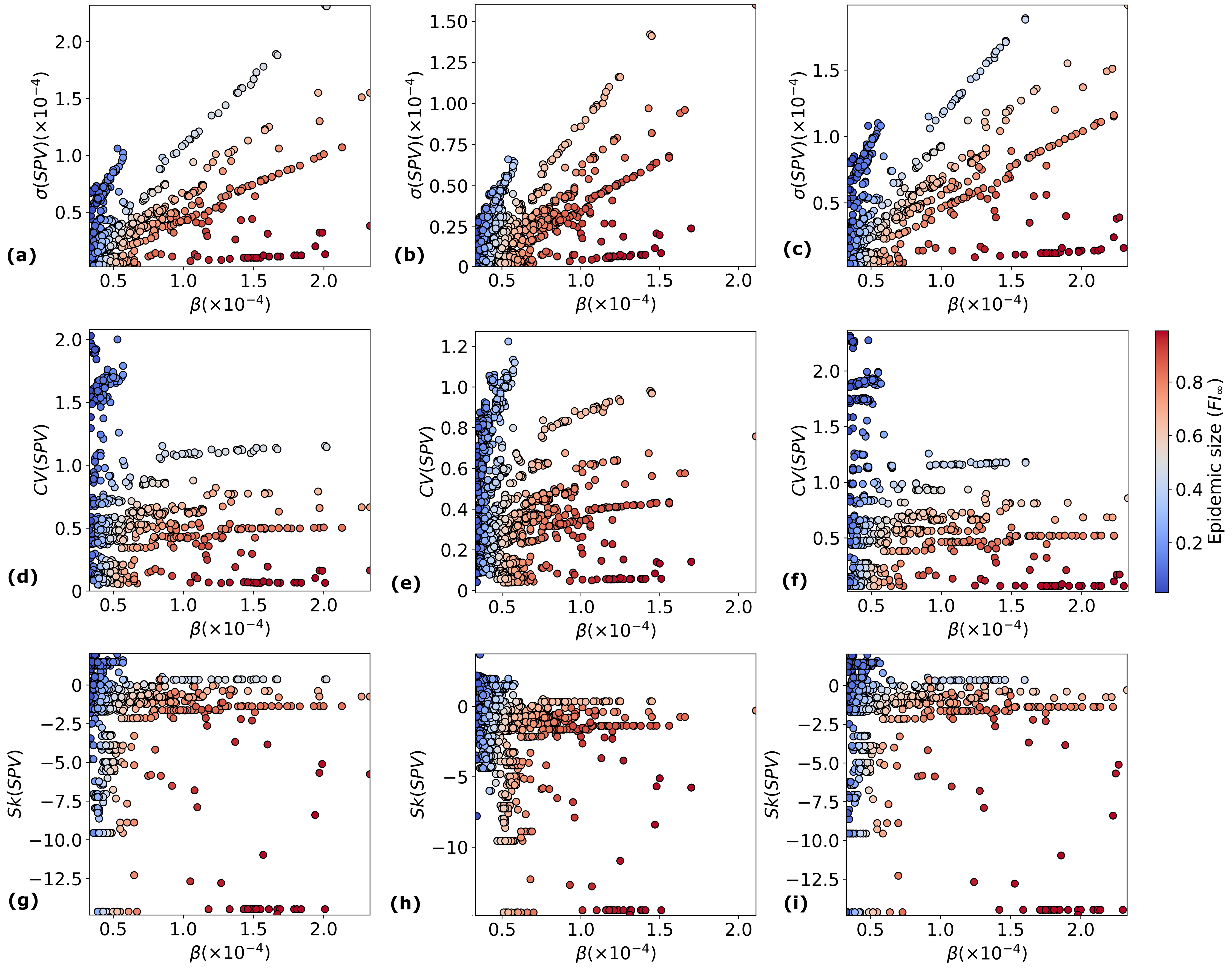

Supplement: S4 Fig — The results presented in the paper use the form si∝1ei (column 1). Two other mathematical forms, si∝1ln(ei+1) (column 2) and si∝1(ei+1)2 (column 3) are explored here, for all 61 ethnicities and 166 viral strains. Only the pairs of SPV characteristics found to have high correlation with FI∞ are shown. (a, b, c) (σ(SPV), β); (d, e, f) (CV(SPV), β); (g, h, i) (Sk(SPV), β). FI∞ is shown as a colourbar. Trends in epidemic size hold across all considered mathematical forms. (TIF) [file pcbi.1006069.s004.tif]
